# Recent advances and clinical relevance of microbiome dynamics in health and disease

**DOI:** 10.1080/19490976.2026.2679197

**Published:** 2026-06-12

**Authors:** Shahin Gavanji, Muhammad Suhail, Elena Bencurova, Thomas Dandekar, Eman M. Othman

**Affiliations:** a Department of Biotechnology, Faculty of Advanced Sciences and Technologies, University of Isfahan, Isfahan, Iran; b Institute of Smart Biomedical Materials, School of Materials Science and Engineering, Zhejiang Sci-Tech University, Hangzhou, China; c School of Pharmacy, Kaohsiung Medical University, Kaohsiung, Taiwan; d Julius-Maximilians-Universität Würzburg, Department of Bioinformatics, Theodor-Boveri-Institute, Biocenter, Würzburg, Germany; e Department of Biochemistry, Faculty of Pharmacy, Minia University, Minia, Egypt; f Julius-Maximilians-Universität Würzburg, Department of Biochemistry-I, Theodor-Boveri-Institute, Biocenter, Würzburg, Germany

**Keywords:** Human microbiome, probiotics, prebiotics, disease, treatment

## Abstract

The human microbiome, comprising trillions of bacteria, viruses, fungi, and archaea, represents an essential partner in human biology rather than a passive collection of microbes. These microbial communities inhabit diverse niches, including the gut, skin, oral cavity, respiratory tract, and urogenital system, where they contribute to digestion, vitamin biosynthesis, immune development, and regulation of host metabolism. Their dynamic interactions form a complex ecosystem that profoundly shapes health across the lifespan. However, with ever increasing reports on the microbiome including perceived health benefits, diagnostic use, detrimental species and immune modulation, we synthesize findings from multiple biomedical fields for this review. It first describes beneficial functions of commensal microbes in maintaining immune tolerance and metabolic balance, then analyzes the effect of diet, geography and medication exposure, the consequences of dysbiosis in gastrointestinal, metabolic, neurological, cardiovascular, autoimmune, and oncological disorders. The article examines the functional potency of the gut microbiome, keystone taxa as well as disease-stage-specific and general dynamics, how microbiomes modulate drug absorption, metabolism, and efficacy, thereby influencing individualized responses to therapy. Furthermore, it evaluates therapeutic approaches, including probiotics, prebiotics, fecal microbiota transplantation, and engineered microbial strategies that seek to restore microbial equilibrium. The significance of this review lies in its integrative perspective, as it links microbiome research to precision medicine, emphasizing that safeguarding microbial diversity is crucial for prevention, early diagnosis, and the personalization of future medical interventions.

In recent years, the study of the human microbiome, defined as the collective genomes of trillions of microorganisms, including bacteria, viruses, fungi, protozoa, and archaea, residing in various niches such as the gut, skin, oral cavity, respiratory tract, and urogenital system, has profoundly reshaped biomedical science. These microbial communities form a complex holobiont within the human host. They have co-evolved to play pivotal roles in metabolism, immunity, and overall physiology.^1^ They contribute fundamentally to essential processes such as the digestion of complex carbohydrates, the biosynthesis of vitamins (e.g., B₁₂, K, folate), the metabolism of xenobiotics, and the maturation of the immune system. Accumulating evidence demonstrates that microbiome research has expanded the horizons of diagnostic, therapeutic, and preventive medicine. The integration of microbiome profiling into personalized healthcare underscores its significance as a determinant of pharmacokinetics and pharmacodynamics, influencing drug metabolism, therapeutic efficacy, and the risk of adverse drug reactions.^2^ ^,^ ^3^ Moreover, integrative metagenomic and metabolomic approaches provide deeper insight into microbial gene functions and metabolic outputs, enabling the identification of disease-specific microbial biomarkers.^3^ Such biomarkers hold great promise for advancing early diagnosis, prognostic evaluation, and patient stratification, ultimately supporting timely and individualized therapeutic interventions. Longitudinal microbiome profiling further enhances this potential by monitoring disease progression and therapeutic responses, thereby strengthening the foundation of precision medicine.^4^ ^,^ ^5^ High-throughput sequencing and multi-omics technologies now permit a comprehensive characterization of microbial communities and the discovery of disease-associated microbial signatures. Notably, alterations in the composition, diversity, or functional capacity of the gut microbiota commonly referred to as dysbiosis have been linked to the development of several complex diseases, including inflammatory bowel disease (IBD), obesity, type 2 diabetes mellitus, and colorectal cancer.^6^

The main aim of this review is to provide readers not only with descriptive accounts of microbial composition but also with a comprehensive overview of how the human microbiome influences health and disease. We critically evaluate the evidence summarizing specific microbial functions and their metabolites in relation to the physiology of healthy individuals and disease pathophysiology, with particular emphasis on gastrointestinal, metabolic, neurological, and oncological disorders. Moreover, the final section is dedicated to microbiome-based therapies, including fecal microbiota transplantation, probiotics, and postbiotics, discussing their mechanisms of action and supporting clinical evidence. Taken together, this review underscores the importance of the dynamic interplay between microbiome and human health and provides a clear, comprehensive resource for researchers and clinicians seeking to harness the microbiome for diagnostic and therapeutic purposes.

In this review, a comprehensive literature search was conducted using multiple electronic databases, including PubMed, Scopus, Web of Science, Google Scholar and ScienceDirect. The search strategy employed various combinations of keywords, including microbiome, useful microbiome, harmful microbiome, microbiome and drug metabolism, microbiome role in disease, and microbiome as therapy. The scope of the search was confined to publications from 1983 to the present (Nov 2025). Two hierarchical sets of criteria governed the selection of sources. The initial, broad criteria encompassed works addressing the general functions and significance of the microbiome. Subsequently, a more stringent set of criteria was applied to identify and include studies specifically focused on the applications of microbiome in contemporary medical practice. This approach ensured a targeted and in-depth synthesis of relevant findings pertaining to the translational potential of microbiome research in modern medicine.

## Health-promoting functions of the human microbiome

The human microbiome plays a vital role in educating both innate and adaptive immunity. This role is particularly pronounced in early life, a critical window during which immune development must establish tolerance, antimicrobial defense, and balanced inflammatory responses.^7^ Far from being a passive collection of microbes, the human body functions as a dynamic ecosystem that hosts a vast and diverse community of trillions of microorganisms collectively referred to as the microbiota. Remarkably, the number of these microbial cells approximates that of human cells, underscoring the deeply interdependent relationship between host and microbiota.^8^ These microbial communities are not evenly distributed across the body. Their composition and abundance vary considerably depending on anatomical location (Table 1). The gastrointestinal tract, especially the colon, represents the most densely populated site. Here, members of the Firmicutes (30%–50% of total population) and Bacteroidetes (20%–30% of total population) phyla dominate.^8^ ^,^ ^9^ These colonizing microbes play indispensable roles in fermenting dietary fibers, synthesizing essential vitamins such as K and B₁₂, and producing short-chain fatty acids (e.g., butyrate) that not only regulate host metabolism but also shape immune function.^9^ Other anatomical sites also host highly specialized microbial communities; each uniquely adapted to local conditions. On the skin, for instance, where the environment is relatively dry and acidic, *Cutibacterium* (e.g., *C. acnes*) may constitute 20%–50% of the microbiota, while *Staphylococcus* species (e.g., *S. epidermidis*) typically represent 20%–40%. These microorganisms contribute to maintaining the skin barrier and compete with potential pathogens, thereby supporting resilience against infection.^10^ The oral cavity harbors its own distinct community, where *Streptococcus* species (20%–40%) are early colonizers, initiating biofilm formation and driving carbohydrate metabolism.^11^ Within the healthy female vaginal tract, *Lactobacillus* species (e.g., *L. crispatus,* and *L. iners*) dominate overwhelmingly, often comprising 70%–90% of the microbiota. Their production of lactic acid establishes a low-pH environment that both sustains health and prevents colonization by harmful microorganisms.^12^ It is essential to acknowledge that these percentages represent dynamic averages rather than static values. Microbial populations continually shift in response to diet, age, genetics, geography, antibiotic exposure, and other environmental factors. Deviations from balanced microbial states, collectively referred to as dysbiosis, are increasingly associated with a wide range of human diseases.^9^

**Table 1.** Human microbiota: composition, abundance, and function.

Body site	Microorganism (genus/species)	Approximate abundance (%)	Properties and functions	References
Gut (colon)	*Bacteroides*	20%–30%	Fermentation of dietary fibers, production of short-chain fatty acids (e.g., butyrate), aid in metabolism, prevention of pathogen colonization	[8]
	*Phylum Firmicutes (e.g. genera Clostridium, Faecalibacterium, Ruminococcus)*	30%–50%	Carbohydrate fermentation, butyrate production, immune system regulation, and maintenance of gut barrier integrity	[8,9]
	*Phylum Actinobacteria (e.g., genus Bifidobacterium spp.)*	2%–10%	Breakdown of complex sugars, production of lactic acid, immune modulation, and anti-inflammatory properties	[8,13]
Skin	*Staphylococcus* (e.g., *S. epidermidis*)	20%–40%	Competition with pathogens (e.g., *S. aureus*), maintenance of acidic pH, and strengthening of the skin's defensive barrier	[14]
	*Cutibacterium* (e.g., *C. acnes*)	20%–50%	The breakdown of sebum (skin oils) and the maintenance of an acidic environment can contribute to acne development under certain conditions	[10,14]
	*Corynebacterium*	10%–20%	Breakdown of organic compounds, competition for nutrients can contribute to body odor	[10]
Oral cavity	*Streptococcus* (e.g., *S. mitis*, and *S. salivarius*)	20%–40%	Primary colonization of dental and mucosal surfaces, sugar metabolism, competition with pathogens, and contribution to plaque formation	[11]
	*Veillonella*	5%–15%	Metabolism of lactic acid produced by other bacteria (microbial symbiosis) helps maintain pH balance	[11]
	*Prevotella*	5%–15%	The breakdown of complex proteins and peptides associated with periodontal diseases occurs when they are present in high abundance	[11,15]
Vagina	*Lactobacillus* (e.g., *L. crispatus*, and *L. iners*)	70%–90%	Production of lactic acid and maintenance of a low pH (3.5–4.5), prevention of pathogen overgrowth and fungal infections	[12]

### Early-life colonization and window of opportunity

Colonization of the human microbiome begins at birth and is profoundly shaped by both the mode of delivery and maternal microbial transfer. Vaginal delivery introduces the newborn to maternal vaginal and gut microbes, such as *Lactobacillus* and *Bacteroides*, thereby initiating the first steps of microbial seeding. In contrast, caesarean delivery often results in delayed colonization, reduced microbial diversity, and altered T-helper cell responses that can persist into early childhood.^16^ ^,^ ^17^ This initial establishment occurs within a critical “window of opportunity” from birth through approximately the first three years of life during which the immune system demonstrates remarkable plasticity.^18^ During this sensitive developmental phase, the microbiome plays a crucial role in guiding immune education, promoting tolerance, and fine-tuning defense against pathogens. Animal studies reinforce these observations, demonstrating that disruptions to microbiome maturation, particularly around the weaning period, can impair the development of peripheral regulatory T cells (T_regs_) and IgA production.

### Pattern recognition and innate immune training

Microbial-associated molecular patterns (MAMPs) including flagellin, lipopolysaccharide, and peptidoglycan interact with pattern recognition receptors (PRRs), such as Toll-like receptors (e.g., TLR5, TLR2), expressed on epithelial and dendritic cells. Through these interactions, PRR signaling guides the education of innate immune cells, stimulates the secretion of mucus and antimicrobial peptides, and fosters the maturation of gut-associated lymphoid tissue (GALT).^19^ ^,^ ^20^ The essential nature of these microbial signals is highlighted in studies of germ-free (GF) mice, which, in the absence of microbial stimulation, exhibit markedly impaired immune development. Such mice display poorly developed Peyer's patches, reduced levels of secretory IgA (sIgA), and underdeveloped intraepithelial lymphocyte populations.^20^ ^,^ ^21^ These findings underscore the indispensable role of the microbiome in shaping not only the structural integrity of the gut's immune landscape but also the broader capacity of the host to mount balanced immune responses.

### Generation of regulatory T cells and immune tolerance

The gut microbiota plays a pivotal role in shaping immune tolerance by driving the differentiation of mucosal FoxP3⁺ regulatory T (T_reg_) cells, including the RORγt⁺ subset that arises in the colon through microbiome-dependent mechanisms. These T_reg_ cells are crucial for suppressing pro-inflammatory Th17 responses, thereby maintaining mucosal homeostasis and preventing harmful inflammation.^22^ ^,^ ^23^ A well-studied example of this interaction is *Bacteroides fragilis*, which produces polysaccharide A (PSA). PSA engages Toll-like receptor 2 (TLR2) on CD4⁺ T cells, triggering T_reg_ expansion and the production of interleukin-10 (IL-10). This pathway promotes immune tolerance toward commensal microorganisms and dietary antigens, reinforcing a harmonious host–microbe relationship.^24^ In addition, microbial metabolites significantly contribute to this immunoregulatory environment. Short-chain fatty acids (SCFAs) particularly butyrate produced during microbial fermentation of dietary fibers, enhance T_reg_ induction and support an anti-inflammatory cytokine milieu.^25^ ^,^ ^26^

### Adaptive immune cell education

Commensal microorganisms play a pivotal role in regulating B-cell class switching toward mucosal IgA within the gut. This process is facilitated through multiple mechanisms, including T-helper cell–dependent pathways and epithelial NF-κB signaling, both of which enhance IgA production. In turn, secretory IgA not only modulates the composition of the gut microbiota but also reinforces immune homeostasis.^27^ ^,^ ^28^ Evidence from both murine and human studies demonstrates that colonization with *Bifidobacterium* during the first week of life is associated with heightened cytokine responses by the age of three, highlighting the enduring influence of early microbial exposure on adaptive immune responsiveness. Moreover, microbial diversity appears to prime Th1 and Th17 effector responses during infection, while regulatory circuits are preferentially engaged under homeostatic conditions to ensure tolerance.^29^ Notably, an overabundance of segmented filamentous bacteria (SFB) can drive robust Th17 activation, which, if left unchecked, may predispose to autoimmune tissue injury.^30^

### Clinical and epidemiological evidence

A recent neonatal cohort study demonstrated that infants exposed to antibiotics shortly after birth exhibited markedly reduced colonization by *Bifidobacterium*, accompanied by diminished antibody responses to pneumococcal and *Haemophilus influenzae* type b (Hib) vaccines at 7 and 15 months of age.^31^ Complementary epidemiological data further support the “biodiversity hypothesis,” which posits that early-life exposure to diverse environmental microbes, such as through soil contact or pet ownership, broadens microbial diversity and, in turn, reduces the incidence of childhood allergies and autoimmune disorders.^32^ ^,^ ^33^

### Cross-organ immune development

Although much of the current literature emphasizes the gut, commensal microbiota residing in other body sites, including the skin, lungs, oral cavity, and vaginal tract, also exert profound influences on local immune regulation. Site-specific microbial interactions foster the induction of tissue-resident immune populations, such as mucosal-associated invariant T (MAIT) cells, which contribute to epithelial repair and facilitate rapid immune responsiveness at barrier surfaces.^34^ Conversely, dysbiosis in these non-intestinal niches can disrupt immune maturation, thereby heightening susceptibility to a range of localized and systemic diseases.^35^

### Keystone microbial taxa across body sites and their role in host health

Beyond overall diversity and taxonomic composition, microbial ecosystems are strongly shaped by keystone taxa—microorganisms that exert a disproportionate influence on community structure, stability, and function relative to their abundance. Keystone taxa act as ecological drivers by modulating nutrient availability, producing key metabolites, shaping interspecies interactions, and reinforcing colonization resistance. Their loss or functional impairment can precipitate community-wide dysbiosis, even when total microbial diversity appears preserved.

#### The vaginal microbiome

Vagina, the microbial organ showing the smallest microbial variation in the human body, is dominant by *Lactobacillus*, which is a species that delays other bacterial colonization that would otherwise results in infections. *Lactobacilli* produces lactic acid, which plays an excellent role in maintaining an acidic pH (<4.5); aiding as a chemical barrier.^36^ Furthermore, *Lactobacillus* spp. form bacteriocins, H2O2, and reactive oxygen species (ROS), delaying the colonization and pathogen's adherence, organisms, which will otherwise cause persistent vulvovaginal infections (RVVI) allied with distress, scent, liberation, sterility, and, if pregnant, could even lead to miscarriages. The vaginal microbiota can be classified into five Community State-Types (CST), indicating various microbial groups.^37^ CST-I has a predominant abundance of *L. crispatus,* CST-II is abundant in *L. gasseri* , CST-III for *L. iners*, and CST-V is a major CTS in *L. jensenii*. Furthermore, CST-IV has a decrease of *Lactobacillus* spp. and higher anaerobic bacteria, including *Prevotella, Atopobium, Sneathia,* and *Gardnerella* that have been linked with vaginosis of bacteria.^38^

#### Skin microbiota

The essential element of the defense system is skin against pathogens. Their anatomical and physiological features change throughout the body, determining microbial composition. A fewer taxa is possessed by skin microbiome compared to other diverse sites of the body because of its textural characteristics, like oil, sebaceous glands, moisture, and acidic pH. External factors are involved in skin microbiome changes, including antibiotics usage, cutaneous burns, hygiene products and skin care, and lifestyle habits. *Staphylococcus, Corynebacterium, Propionibacterium*, and *Streptococcus* are the most dominant skin genus. Furthermore, *Propionibacterium* species (lipophilic) are present abundantly in oilier sites, whereas *Staphylococcus* and *Corynebacterium* species thrive in humid niches.^39^ ^,^ ^40^

#### The eye microbiota

The three major microbial niches of the eye are: the eyelid skin, meibum, and conjunctiva, which fluctuate a lot in composition and diversity. Alike to the skin microbiome, the microbial composition of the eyelids is subjugated mainly by *Staphylococcus* and *Propionibacterium*. *Propionibacterium* and *Pseudomonas* are present highly in the meibum, while *Propionibacterium* defines the conjunctiva exclusively. The dysbiosis in the conjunctiva microbiome has been linked with diverse health conditions, including mucosa- associated lymphoid tissue (MALT) lymphoma, keratoconjunctivitis, and high glucose levels on the ocular surface due to diabetes mellitus. Notably, the conjunctional microbiota in MALT lymphoma patients is subjugated through *Delftia.* ^41^ The mice studies revealed a decline in Type 2 diabetes diversity during the evaluation role of the ocular microbiota linked to diabetes mellitus, whereas a rise in *Bacteroides* and a reduction in *Acinetobacter* and *Proteobacteria* are detected in Type 2 diabetes (T2D).^42^ ^,^ ^43^

#### The ear microbiota

The microbiota of the ear canal is almost same as skin. Hence, *Staphylococcus*, *Corynebacterium*, and *Propionibacterium* genera are predominant taxa. Still a debate is there over whether the middle ear is colonized by nasopharynx microbes or if this is a sterile site. Although no microbial colonization was reported by the previous findings, a recent Illumina microbiome profiling study reported that *Actinobacteria*, *Firmicutes* and *Proteobacteria* are colonized in the middle ear.^44^ Otitis media infections can be categorized as Acute Otitis Media (AOM) or Chronic Otitis Media with Effusion (COME). Children and adults who suffer from Otitis Media inflammation are surrounded by dysbiotic changes. The development and pathogenesis of AOM are reliant on the microbiome of the nasopharynx, with *Alloiococcus*, *Haemophilus*, *Turicella*, *Staphylococcus*, *Moraxella*, and *Streptococcus* being taxa allied commonly with this condition.^45^

#### The oral microbiota

The most diverse body niches are the oral microbiome, preceded only with the colon. A diversity can be seen highly in the oral cavity because of its many structural and biological niches protecting a plethora of several communities of the microbes. These niches include oral mucosa, tongue, saliva, soft and hard tissues, and the teeth's surface. Each surface contains distinctive communities; thus, it supplies the nutrients and conditions needed for these distinctive microbes. For example, the tongue flora is different from that in plague or the hard tissues of the oral cavity because of its specific microenvironment. Several oral diseases, including dental cavities, periodontitis, gingivitis, and tooth loss, subsequently, are developed by both fungal and bacterial communities. The composition of bacteria mainly contains *Bacteroidetes, Firmicutes, Proteobacteria*, *Spirochaetes, Fusobacteria*, and *Actinobacteria*. *Gemella*, *Streptococcus*, *Abiotrophia*, *Rothia*, *Granulicatella*, *Neisseria*, and *Prevotella*, are the most dominant bacterial taxa among the bacterial taxa in the oral cavity.^46^ The fungal flora often contains in high abundance *Candida*, *Cladosporium*, *Saccharomyces Aureobasidium*, *Fusarium*, *Aspergillus*, and *Cryptococcus.* ^47^ The fungi are commensals as long as the immune status of the person is strong.

#### The microbiota of the gastrointestinal tract

The most diverse and largest reservoir of all the human body niches is the gastrointestinal microbiome. A specific environment is provided by each digestive organ part from mouth to the anal cavity, which allows the colonization and growth of organisms. *Proteobacteria*, *Actinobacteria*. *Firmicutes*, and *Bacteroidetes*, are the most common phyla across the gut tube. *Veillonella*, *Streptococcus*, and *Prevotella* are the most common dominant bacterial taxa in the esophagus. *Proteobacteria* and *Firmicutes* dominate the stomach microbial communities. Many studies reported that *Helicobacter pylori*, found in the stomach, is the part of the normal flora, which was lost through modern lifestyles. Other studies report that positive *H. pylori* status is correlated with high relative abundance of non-*Helicobacter* bacteria from the *Proteobacteria*, *Spirochaetes*, and *Acidobacteria* phyla, besides a reduced abundance of *Actinobacteria*, *Bacteroidetes*, and *Firmicutes*. Furthermore, *H. pylori* is not only a causative agent of gastritis but also linked with gastric cancer. Some studies reported that the risk of celiac disease, asthma and esophageal adenocarcinoma could be lowered by *H. pylori* infections. The microbial dysbiosis resulted in Gastroesophageal reflux disease (GERD), Barrett's esophagus, and esophageal carcinoma.^48^ Persistent GERD that progresses to Barrett's esophagus, prompting to esophageal carcinoma, has been linked to a rise of *Fusobacterium*, *Veillonella*, and *Prevotella*, taxa, which are not existing in healthy individuals. An environment contained high concentrations of oxygen and antimicrobials alongside with a short transit time, which allows the fast growth of facultative anaerobes, characterized by the small intestine. A 90% energy of the host is absorbed from the diet and is divided into three parts: duodenum, jejunum, and ileum. The *Firmicutes*, *Actinobacteria*, and *Proteobacteria*, taxa that subsidize mostly to nutrient digestion, such as lipids, protein, and simple sugars, are the high abundant phyla in the duodenum.^49^ ^,^ ^50^

Moreover, there are also other important factors which can shape the gut microbial composition. As summarized in Table 2, diet, geography, and medication exposure tightly interfere with external environmental and dietary factors.

**Table 2.** Effects of diet, geography, and medication exposure on gut microbiome composition and function.

Determinant	Population/context	Key microbial changes (taxa/functions)	Functional consequences	Clinical/biological implications	Representative references
Dietary fiber-rich (plant-based, traditional diets)	Rural Africa, agrarian societies	↑ *Prevotella*, ↑ SCFA-producing Firmicutes (*Faecalibacterium*, *Roseburia*)	↑ Butyrate & propionate production; enhanced carbohydrate fermentation	Improved gut barrier integrity; reduced inflammatory and metabolic disease risk	[51,52]
Western diet (high fat, refined sugars)	North America, Europe	↑ *Bacteroides*, ↑ *Bilophila wadsworthia*, ↓ microbial diversity	↑ LPS, secondary bile acids; ↓ SCFA production	Increased risk of obesity, T2D, IBD, CRC	[53,54]
High animal protein intake	Industrialized populations	↑ *Alistipes*, ↑ *Bacteroides*; ↑ sulfur-reducing bacteria	↑ Hydrogen sulfide; altered bile acid metabolism	Mucosal inflammation; CRC risk	[55,56]
Geography (industrialized vs non-industrialized)	Urban vs rural populations	↑ *Bacteroides* (industrialized); ↑ *Prevotella*, ↑ diversity (non-industrialized)	Distinct polysaccharide utilization pathways	Differential immune maturation and metabolic profiles	[57]
Antibiotic exposure (broad-spectrum)	All age groups	↓ Diversity; ↑ Proteobacteria (*Enterobacteriaceae*); loss of keystone taxa	↓ SCFAs; ↓ colonization resistance	*C. difficile* infection; long-term immune dysregulation	[58]
Early-life antibiotics	Neonates, infants	↓ *Bifidobacterium*; delayed microbiome maturation	Impaired immune education	Increased allergy, asthma, obesity risk	[59]
Proton pump inhibitors (PPIs)	Long-term users	↑ Oral taxa in gut; ↑ *Enterococcus*, ↑ Proteobacteria	Altered gastric barrier; ↑ bacterial translocation	Increased infection risk, SIBO	[60]
Metformin therapy	Type 2 diabetes patients	↑ *Akkermansia muciniphila*, ↑ *Escherichia* spp.	Altered glucose metabolism; ↑ SCFAs	Improved insulin sensitivity; GI side effects	[61]
Statins	Cardiovascular patients	↑ *Lactobacillus*, ↑ bile acid-modifying bacteria	Altered bile acid pools	Modified lipid response to therapy	[62]
Geography-specific sanitation & lifestyle	Low- vs high-hygiene environments	↑ environmental taxa; ↑ microbial richness	Enhanced immune tolerance	Reduced allergy and autoimmune prevalence	[63,64]

### Health risks associated with the human microbiome

The human microbiome, comprising trillions of microorganisms that inhabit the gut, skin, oral cavity, and other body sites, plays a crucial role in digestion, metabolism, and immune regulation (Table 3). Dysbiosis, defined as a disruption of the balanced microbial ecosystem, can compromise mucosal barrier integrity, thereby promoting inflammation and immune dysregulation.^65^ Altered gut microbial composition has been strongly implicated in inflammatory bowel diseases, including Crohn's disease and ulcerative colitis.^66^ Similarly, metabolic disorders such as obesity, type 2 diabetes, and metabolic syndrome have been associated with microbial imbalance, in part reflecting Western dietary patterns and shifts in microbiota-derived metabolites.^67^ ^,^ ^68^ Dysbiosis is further linked to oxidative stress and chronic inflammation through excessive production of reactive oxygen species.^69^ It also contributes to gastrointestinal motility disorders, such as small intestinal bacterial overgrowth, by impairing epithelial barrier function and perturbing immune pathways. Importantly, altered microbial metabolites have been shown to promote carcinogenesis, particularly in colorectal cancer, through genotoxic effects and pro-inflammatory signaling.^70^ ^,^ ^71^ A consistent finding in colorectal cancer patients is the depletion of butyrate-producing bacteria, which are essential for maintaining colonocyte health and exert tumor-suppressive effects.^72^ Beyond the gut, dysbiosis influences systemic physiology. Disruptions in the gut–brain axis have been associated with neuropsychiatric disorders, including anxiety and depression, via alterations in neurotransmitter synthesis and immune signaling.^73^ Reduced microbial diversity also predisposes to opportunistic infections, notably *Clostridioides difficile*, especially following antibiotic use.^74^ Moreover, microbial imbalances have been implicated in extra-intestinal conditions such as acne, psoriasis, and periodontitis.^75^ Environmental factors, including low-fiber diets, food additives, antibiotic exposure, and sedentary lifestyles, are key drivers of dysbiosis.^76^ Although therapeutic interventions aimed at restoring microbiome equilibrium, including probiotics, prebiotics, dietary modification, and fecal microbiota transplantation, show promise,^77^ the causal relationships between dysbiosis and disease remain complex and often bidirectional. Continued research is therefore required to disentangle these mechanisms and to optimize microbiome-targeted therapies.^78^

**Table 3.** Microbiome alterations and their role in human diseases.

Disease group	Key microbial changes	Involved metabolites	Pathogenic mechanisms	References
Obesity	↑ Firmicutes/Bacteroidetes ratio; ↑ *Bilophila wadsworthia*	Acetate, SCFAs	Enhanced energy harvest; acetate-driven lipogenesis; pro-inflammatory taxa promoting intestinal inflammation	[79‐81]
Type 2 diabetes (T2D)	↓ *Akkermansia muciniphila*; ↑ *Prevotella copri*	LPS, Branched-chain amino acids (BCAAs)	Increased gut permeability → endotoxemia; TLR4 activation → chronic low-grade inflammation; impaired insulin signaling	[82‐84]
Non-alcoholic fatty liver disease (NAFLD)	↑ *Escherichia coli*, ↑ *Clostridia*, ↑ Enterobacteriaceae	Ethanol, TMA/TMAO	Microbial ethanol damages hepatocytes; TMA → TMAO promotes steatosis and inflammation.	[85,86]
Inflammatory bowel disease (IBD)	↓ *Faecalibacterium prausnitzii*; ↑ Proteobacteria (esp. *E. coli*)	SCFAs (butyrate ↓), LPS	Loss of butyrate → impaired barrier + T_reg_ function; LPS-driven NF-κB activation → chronic inflammation	[87‐92]
Irritable bowel syndrome (IBS)	↓ *Lactobacillus*, ↓ *Bifidobacterium*; ↑ Firmicutes/Bacteroidetes ratio	Methane, Hydrogen sulfide, Serotonin (5-HT)	Gas metabolism affects motility; microbial regulation of serotonin alters gut–brain axis.	[93‐95]
Colorectal cancer (CRC)	↑ *Fusobacterium nucleatum*, ↑ ETBF (*B. fragilis*), ↑ *E. coli* (pks+)	Colibactin, BFT toxin, ROS	DNA damage (colibactin); NF-κB & IL-17 activation; chronic inflammation; β-catenin signaling activation	[96,97]
Parkinson's disease (PD)	↓ *Prevotella*; ↑ Enterobacteriaceae	SCFAs (butyrate ↓), LPS	Reduced barrier function; systemic inflammation; *α*-synuclein misfolding & propagation via vagus nerve	[8,98]
Alzheimer's disease (AD)	↑ *Escherichia/Shigella*; ↓ *Bifidobacterium*, ↓ *Eubacterium rectale*	LPS, Bacterial amyloids, TMAO	Neuroinflammation; amyloid-β aggregation; tau hyperphosphorylation; impaired T_reg_ control	[99]
Multiple sclerosis (MS)	↑ *Akkermansia muciniphila*, ↑ *Methanobrevibacter smithii*; ↓ *Parabacteroides distasonis*	SCFAs (propionate ↓)	Reduced T_reg_ expansion; increased Th17 activation; autoimmunity exacerbation	[58,100‐104]
Depression/mood disorders	↓ *Lactobacillus*, ↓ *Bifidobacterium*; ↑ pro-inflammatory taxa	GABA, Serotonin precursors, Kynurenine metabolites	Reduced neurotransmitter production; cytokine-driven neuroinflammation; neurotoxic kynurenine pathway	[58,99‐105]
Autism spectrum disorder (ASD)	↑ *Clostridium bolteae*, ↑ *Desulfovibrio*; ↓ *Bifidobacterium*, ↓ *Prevotella*	p-Cresol, Propionic acid	Neurotoxic metabolites impair mitochondrial & neuronal function; gut–brain axis disruption	[106]
Atherosclerosis	↑ Enterobacteriaceae, ↑ Clostridiaceae	TMA/TMAO, Indoxyl sulfate	TMAO → foam cell formation; vascular inflammation; endothelial dysfunction	[107‐109]
Hypertension	↓ SCFA-producing bacteria (*Lactobacillus, Bifidobacterium*)	SCFAs, LPS	Loss of vasodilatory SCFAs; ↑ endotoxin → vascular inflammation	[110,111]
Autoimmune diseases (RA, T1D, SSc)	↓ SCFA producers (*Faecalibacterium prausnitzii, Roseburia*); ↑ *Bacteroides*, ↑ *Prevotella*	SCFAs (butyrate ↓), LPS	Impaired T_reg_ expansion; enhanced Th17 responses; molecular mimicry; “leaky gut” increasing antigen translocation	[96,112‐114]

### Threats and risks resulting from disruption of the gut microbiome

The gut microbiome represents one of the most complex and diverse ecosystems in the human body, with more than 70% of immune cells residing in proximity to it. Perturbations in this ecosystem can enable pathogenic or opportunistic bacteria to proliferate unchecked. A notable example is the overgrowth of *Clostridioides difficile*, which is associated with severe diarrhea, colitis, and, in some cases, fatal outcomes. Alterations in microbial composition have also been strongly linked to inflammatory bowel diseases (IBD), including Crohn's disease and ulcerative colitis. In these conditions, increased intestinal permeability permits the translocation of microbial toxins and antigens into the circulation, thereby driving systemic inflammation and contributing to autoimmune pathology. Microbial dysbiosis is further implicated in metabolic disorders such as obesity, type 2 diabetes, and metabolic syndrome, in part due to the enhanced capacity of certain microbial populations to extract energy from dietary substrates. Disruptions in microbiota composition can also diminish the production of short-chain fatty acids, which are critical for maintaining intestinal epithelial integrity and immune homeostasis. Collectively, these changes establish a cycle of chronic inflammation, metabolic dysregulation, and heightened susceptibility to chronic disease.^115-117^

### Effects of the microbiome on the brain and mental health (gut–brain axis risks)

The gut–brain axis represents one of the most compelling frontiers in contemporary biomedical research.^118^ The intestinal microbiome has been shown to influence behavior, mood, and cognitive function through several mechanisms, including the synthesis of neurotransmitters such as serotonin and γ-aminobutyric acid (GABA), as well as modulation of immune signaling pathways. Disruption of this bidirectional network has been linked to an elevated risk of neuropsychiatric and neurodegenerative conditions, including depression, anxiety, autism spectrum disorders, Alzheimer's disease, and Parkinson's disease. For instance, the depletion of anti-inflammatory bacterial taxa can exacerbate neuroinflammation and impair synaptic function. At the same time, shifts in microbial composition may alter stress-response pathways, thereby increasing vulnerability to anxiety. Compelling experimental evidence underscores this connection: fecal microbiota transplantation from individuals with depression into germ-free animals has been shown to induce depression-like behaviors in the recipient hosts. Such findings highlight that dysbiosis extends far beyond gastrointestinal pathology and may exert profound effects on central nervous system function. Although the field remains in its early stages, current evidence strongly suggests that preserving microbiome health may be as critical for human well-being as safeguarding cardiovascular or pulmonary function.^72^ ^,^ ^106^ ^,^ ^119^

### Risks of antibiotics and medical interventions on the microbiome

While antibiotics remain indispensable for controlling infectious diseases, their widespread or prolonged use poses a significant threat to the integrity of the microbiome. Overexposure can eliminate beneficial bacterial populations, thereby creating ecological niches for pathogenic organisms to proliferate.^58^ Such perturbations may persist for weeks, months, or even years, with early-life exposures being particularly consequential due to the ongoing maturation of the infant microbiome. Beyond antibiotics, interventions such as chemotherapy, radiotherapy, and gastrointestinal surgeries can similarly disrupt microbial ecosystems. Environmental factors also play a critical role: the extensive use of disinfectants and antibacterial products reduces exposure to benign microbes, thereby diminishing overall microbial diversity, a change that has been linked to an increased prevalence of allergies, asthma, and autoimmune disorders. Infant feeding practices further influence microbial composition; formula feeding, in contrast to breastfeeding, can lead to distinct microbiota profiles that may predispose to future immune dysfunction. Collectively, these observations underscore the need to pair medical interventions with strategies aimed at preserving and restoring microbiome health.^102-104^

### Long-term consequences of microbiome disruption and future risks

If disruptions to the microbiome are not promptly addressed, they can have enduring effects on individual health and may even influence subsequent generations.^99^ ^,^ ^105^ Evidence suggests that the maternal microbiome during pregnancy influences the immune development and metabolic programming of the offspring. Conversely, reduced microbial diversity observed in modern urban populations may contribute to the rising prevalence of chronic diseases in the 21st century. Beyond well-characterized conditions, other risks remain incompletely understood; For instance, alterations in microbiome composition may modify the body's sensitivity to environmental toxins or pharmaceuticals, thereby increasing the likelihood of adverse reactions or toxicities. Emerging studies also suggest that an imbalanced microbiome can influence aging processes and potentially reduce lifespan. Additionally, the proliferation of antibiotic-resistant microorganisms, which can be transmitted through the food chain or interpersonal contact, represents a significant public health concern. Coupled with contemporary lifestyle factors, including high consumption of processed foods, sedentary behavior, and environmental pollution, these microbiome-related vulnerabilities are likely to become increasingly pronounced. Collectively, these observations underscore that preventive strategies to preserve microbial diversity and ecosystem balance are essential for long-term health, rather than optional.^3^ ^,^ ^120^

### Functional potency of the gut microbiome

Over recent decades, research has focused on gut microbiome alterations in disease. The gut microbiome contributes to metabolic disorders, including obesity, nonalcoholic fatty liver disease (NAFLD), and type 2 diabetes. Changes in lifestyle, such as reduced physical activity and energy-dense Western diets, are linked to obesity and associated with compositional shifts such as increased *Mollicutes* (*Firmicutes.* ^121^). Obesity-related microbiomes show enhanced energy harvest, reduced communities of *Oscillibacter, Alistipes, Akkermansia*, and *Faecalibacterium*, and characteristic metabolite changes. Furthermore, NAFLD patients display higher *Bacteroidetes* and reduced short-chain-fatty-acid–producing and 7α-dehydroxylating *Firmicutes.* ^122^ ^,^ ^123^ Microbiota-transfer experiments show that communities from hyperglycemic donors promote macrovesicular steatosis under a high-fat diet, whereas normoglycemic donor microbiota induce only mild steatosis, underscoring the microbiome's role in NAFLD susceptibility.^124^ In children, NAFLD and particularly nonalcoholic steatohepatitis are associated with reduced α-diversity and enrichment of genes encoding inflammatory bacterial products, suggesting that dysbiosis contributes to pathogenesis and may serve as a biomarker of disease presence and its severity.^125^

​Because of its close crosstalk with the immune system, gut dysbiosis is linked to a broad spectrum of inflammatory conditions.^126^ This opens possibilities to use microbiome profiles for patient stratification and to target microbial communities or their metabolic outputs therapeutically. Achieving this requires deeper functional profiling (e.g., metagenomics, metatranscriptomics) to define how microbial genes, pathways, and metabolites shift across health and disease.^127^ The gut, harboring the highest microbial density and continuous mucosal immune exposure, is central to autoimmune and inflammatory diseases, including inflammatory bowel disease (IBD), type 1 diabetes, colorectal cancer, and metabolic syndrome, and influences extra-intestinal conditions such as rheumatic disease, allergy, atopy, and skin inflammation.^128-130^

​Inflammatory bowel diseases (IBDs), including Crohn's disease and ulcerative colitis, reflects a dysregulated immune response to commensal microbiota on a strong genetic background.^131^ Genome-wide association studies have identified many risk loci, but functional mechanisms are well characterized for only a few genes, such as NOD2 and IL23R, which directly regulate microbial handling. Integrating microbial functional data with host genetics may clarify their in vivo roles. Environmental and nutritional factors modulate this axis: low vitamin D, which regulates pro-inflammatory signaling and T-regulatory cell development, increases Crohn's disease risk and is partly shaped by microbiome-dependent vitamin D receptor regulation, while dietary fiber, metabolized into anti-inflammatory short-chain fatty acids (SCFAs), protects against IBD.^131^ ^,^ ^132^ Reduced diversity in IBD likely reflects functional shifts, including expansion of inflammation-tolerant Enterobacteriaceae and loss of SCFA-producing *Clostridia*, reinforcing a pro-inflammatory milieu. These host–microbe and microbe–microbe feedback loops are promising targets for drugs, probiotics, and interventions aimed at redox metabolism and other key microbial processes.^133-135^

​Neurodevelopmental disorders such as autism spectrum disorder (ASD) and attention deficit hyperactivity disorder (ADHD) share behavioral deficits and have been linked to altered hypothalamic–pituitary–adrenal (HPA) axis responses and microbiome-associated metabolic changes.^136^ Gastrointestinal symptoms are common in ASD, and affected children show distinct microbial and metabolite profiles. Ming et al. reported altered amino acid metabolism and increased oxidative stress in ASD, with a subgroup displaying distinct gut microbiota compared with controls.^137^ ASD children often exhibit increased *Proteobacteria* and *Bacteroidetes* and reduced *Firmicutes* and *Bifidobacteria*, with more *Clostridia* and fewer *Prevotella* in those with gastrointestinal disturbances.^123^ ^,^ ^124^ Sandler et al. proposed that disruption of indigenous microbiota in some children favors colonization by neurotoxin-producing bacteria, coinciding with onset or worsening of autistic features after broad-spectrum antibiotic exposure and chronic diarrhea.^138^ In summary, we can confirm not only diagnostic changes in microbiota composition signifying a certain disease state, but there is an intricate connection between microbiome and host, altered metabolites and inflammatory conditions from changes in the microbiome are influencing also disease states.

### Microbiome and drug metabolism

The human gut microbiome constitutes a dense and metabolically active ecosystem that interacts continuously with both orally administered and systemic drugs (Table 4). Gut microbes express enzymes capable of directly modifying pharmaceutical compounds, generating metabolites that may be inactive, active, or even toxic.^139^ Beyond these direct transformations, the microbiome can influence host drug-metabolizing enzymes and transporters, thereby modulating drug absorption, distribution, and clearance.^140^ Inter-individual differences in microbial composition and gene content thus contribute significantly to variability in drug exposure, therapeutic efficacy, and adverse reactions among patients. Systematic screens have revealed that numerous commonly used oral drugs are substrates for bacterial metabolism, indicating that microbiome-mediated effects on pharmacokinetics are widespread rather than exceptional.^139^ These microbial activities can exert both local effects within the gut, altering luminal drug concentrations and local toxicity, as well as systemic effects by modifying circulating levels of parent compounds and their metabolites.^141^ Classic examples, such as bacterial inactivation of the cardiac glycoside digoxin and microbiome-mediated activation or reactivation of chemotherapeutic metabolites, established the foundational principle that gut microbes can substantially alter drug responses.^141-143^ More recently, high-throughput studies have mapped hundreds of drug–bacterium interactions, linking specific bacterial genes to defined chemical transformations.^141-146^ Collectively, these findings underscore the necessity of integrating microbiome biology into ADME (absorption, distribution, metabolism, and excretion) considerations for drug development and precision medicine.^147^

### Mechanisms: how microbes change drug metabolism (direct and indirect routes)

Microbial drug metabolism occurs through both direct enzymatic transformations and indirect modulation of host pathways.^148^ ^,^ ^149^ Direct mechanisms include reduction, hydrolysis, deconjugation, such as bacterial β-glucuronidases removing glucuronide conjugates, deamination, nitroreduction, and complex redox reactions that modify molecular scaffolds.^150^ Enzymes encoded in bacterial genomes, often lacking homology to well-characterized host drug-metabolizing enzymes, can catalyze metabolic reactions that are chemically distinct from hepatic cytochrome P450 activity. In particular, anaerobic gut bacteria perform reductions that host hepatic enzymes cannot.^143^ ^,^ ^151^ Indirect mechanisms involve microbiome-derived small molecules, including short-chain fatty acids, secondary bile acids, and aromatic metabolites that modulate host nuclear receptors and transcriptional programs, thereby influencing the expression and activity of phase I and phase II drug-metabolizing enzymes and transporters (e.g., CYPs, UGTs, ABC transporters).^143^ ^,^ ^147^ ^,^ ^148^ Microbial communities also modify intestinal barrier properties, gastrointestinal transit time, and luminal pH, all of which affect drug dissolution, absorption, and exposure to microbial metabolism.^151^ ^,^ ^152^ Additionally, microbes may sequester or bioaccumulate drugs, reducing luminal bioavailability.^141^ ^,^ ^153^ Importantly, microbial metabolic activity is often determined by specific genes rather than taxonomic identity; thus, individuals with similar species composition may differ substantially in drug metabolism if their microbial gene repertoires diverge.^3^ ^,^ ^149^ ^,^ ^154^ This gene-centric perspective enables predictive models that leverage metagenomic gene abundance to forecast metabolic potential for particular drugs. Experimental studies have causally linked specific microbial genes to metabolite formation and demonstrated systemic pharmacokinetic consequences in animal models.^17^ ^,^ ^142^ ^,^ ^149^

### Drug metabolism mechanisms by the microbiome: a mechanistic and systems-level perspective

The human gut microbiome encompasses a network of reductive and hydrolytic biochemical pathways that complement hepatic Phase I and II metabolism, which is primarily oxidative and conjugative. This microbial capacity enables the activation, inactivation, detoxification, and even re-toxification of xenobiotics, exhibiting substantial interindividual variability, specifically at the bacterial strain level.^142^ ^,^ ^155^ Systematic screening of 271 orally administered drugs across 76 representative gut bacterial species demonstrated that a considerable proportion of drugs undergo direct chemical modification by microbial enzymes, and these transformations can be predicted based on the strains' metabolic gene content.^155^ Comprehensive reconstruction of metabolic pathways across over 7,300 human microbial strains further revealed that at least 98 drugs are targets of strain-specific microbial reactions, with heterogeneous distribution across microbial lineages, laying the groundwork for microbiome-informed precision pharmacology.^142^ At the biochemical level, microbial reactions include hydrolysis (β-glucuronidases, sulfatases, esterases), reductive reactions (azoreductases, nitroreductases, carbonyl reductases), specific decarboxylations and dehydroxylations, and enterohepatic deconjugations.^139^ For example, microbial β-glucuronidases (GUS) cleave glucuronide conjugates, thereby reactivating excreted inactive metabolites. A clinically significant instance is the reactivation of SN-38G (the irinotecan conjugate) in the intestinal lumen, which contributes to chemotherapy-induced colitis.^143^ Structural diversity in GUS active-site pockets and loop regions underlies interindividual variability in both the spectrum of drugs susceptible to deglucuronidation and the associated toxicity. Microbial sulfatases degrade xenobiotic and endobiotic sulfate esters, including hormones and bile acids, thereby influencing enterohepatic circulation and drug bioavailability. Esterases and glycosidases contribute to the deglycosylation and hydrolysis of prodrugs, potentially overriding host-controlled release of active moieties. In the anaerobic colonic environment, reductases are abundant: azoreductases cleave ─N═N─ bonds, converting azo prodrugs (e.g., sulfasalazine, balsalazide, olsalazine) into active 5-aminosalicylic acid (5-ASA) and aromatic carriers^156^; nitroreductases reduce –NO₂ groups to amines or hydroxylamines, mediating both drug activation and detoxification.^139^ Strain-specific examples include the reduction of digoxin to dihydrodigoxin by *Eggerthella lenta*, encoded by the *cgr* operon; *cgr* expression is induced by digoxin and inhibited by arginine.^99^ Decarboxylation and dehydroxylation pathways are also critical. *Enterococcus faecalis* decarboxylates L-dopa to dopamine via tyrosine decarboxylase (*tdc*), and *E. lenta* subsequently dehydroxylates the catechol ring to form m-tyramine, reducing drug delivery to the brain. Other microbial reactions, including dealkylation, diamination, and aromatic-ring cleavage, affect xenobiotic polarity, permeability, and host Phase II metabolism. Microbial pathways that influence enterohepatic circulation, such as GUS, sulfatases, and bile-salt hydrolases, can alter drug reabsorption, half-life, and plasma fluctuations, directly impacting dosing and scheduling.^3^ ^,^ ^140^ Additionally, some bacteria sequester or bioaccumulate drugs, reducing luminal availability.^157^ Given the tight interconnection between host metabolic networks, diet, and the gut environment, accurate prediction of drug responses requires consideration of strain- and molecule-level microbial interactions. Clinically, these mechanisms have been demonstrated to be of significance. Irinotecan (CPT-11), following conjugation to SN-38G, is reactivated by gut β-glucuronidases, causing colitis; selective GUS inhibition mitigates toxicity without compromising antitumor efficacy.^143^ ^,^ ^158^ Digoxin inactivation by *E. lenta* suggests that the presence of the *cgr* gene may necessitate dose adjustment or closer monitoring, as dietary arginine can inhibit this pathway.^156^ Azo prodrugs for IBD, such as sulfasalazine and balsalazide, are primarily activated by bacterial azoreductases, and strain-level variability contributes to heterogeneous clinical responses. L-dopa in Parkinson's disease is activated through microbial decarboxylation and dehydroxylation, remaining active despite host enzyme inhibition by carbidopa. Microbiome-mediated interactions can also affect drug safety indirectly, as illustrated by sorivudine–5-fluorouracil interactions and statin responses influenced by microbial bile acid composition. Collectively, these findings establish a foundation for personalized microbiome pharmacogenomics, where stool sampling, strain-level screening, and systems modeling can quantify an individual's microbial metabolic capacity for specific drugs and guide optimal dosing strategies.^157^

**Table 4.** Microbiome alterations and their role in human diseases.

Drug/compound	Microbial modification (including microbes)	Biochemical pathway (enzyme)	Clinical consequence	References
Digoxin	Inactivation of digoxin to dihydrodigoxin by *Eggerthella lenta*	cgr operon (cardiac glycoside reductase)	Reduced drug efficacy; variability depending on microbial gene presence; inhibited by dietary arginine	[99,156]
Irinotecan (SN-38G conjugate)	Reactivation of SN-38G in intestine by gut bacteria (*E. coli*, *Clostridium* spp.)	β-glucuronidase (GUS)	Chemotherapy-induced colitis; toxicity mitigated by GUS inhibitors	[143,145,158]
Sulfasalazine/Balsalazide/Olsalazine	Cleavage of azo bonds to release active 5-ASA by *Clostridium spp.* and *Bacteroides spp.*	Azoreductases	Activation of prodrug in IBD therapy; interindividual variability in response	[145,156]
L-Dopa	Decarboxylation of L-Dopa to dopamine by *Enterococcus faecalis*; dehydroxylation to m-tyramine by *Eggerthella lenta*	Tyrosine decarboxylase (tdc); dehydroxylase	Reduced L-dopa delivery to the brain in Parkinson's disease; resistance to host carbidopa inhibition	[159]
Sorivudine + 5-Fluorouracil	Sorivudine metabolism by gut bacteria generates a toxic metabolite inhibiting 5-FU degradation	Microbial nucleoside metabolism	Severe toxicity due to drug–drug–microbiome interaction	[160‐162]
Statins	Modulation of statin activity through bile acid metabolism by *Lactobacillus spp.*, *Bifidobacterium spp.*, and others	Bile salt hydrolases, deconjugation enzymes	Altered plasma levels and therapeutic efficacy	[163,164]

### Methods and experimental approaches used to assess microbiome drug interactions

Researchers employ complementary approaches to investigate microbiome–drug interactions, with integrative strategies yielding the most robust causal inferences. High-throughput *ex vivo* screens test panels of isolated gut strains or subject-specific fecal communities against drug libraries, using mass spectrometry to detect resulting chemical transformations.^73^ ^,^ ^141^ Functional genomics approaches, including transposon mutagenesis, heterologous expression, and comparative genomics, map enzymatic functions to specific microbial genes.^165^ Gnotobiotic mouse models, encompassing germ-free and defined-community animals, enable causal *in vivo* studies by colonizing mice with exact strains or engineered mutants and measuring drug and metabolite levels in plasma and tissues to quantify microbial contributions.^99^ ^,^ ^166^ Pharmacokinetic modeling that integrates data from germ-free, mono-associated, and conventionally colonized animals can disentangle host and microbial sources, even when both produce similar metabolites.^99^ ^,^ ^141^ Human studies typically combine paired fecal incubations, metagenomic profiling, and targeted metabolomics, generating mechanistic hypotheses that often require experimental validation.^167^ Advanced *ex vivo* platforms, such as continuous fermentation systems and gut-on-a-chip models, allow controlled, physiological simulation of luminal conditions. Multi-omics approaches, including metagenomics, metatranscriptomics, metaproteomics, and metabolomics, facilitate the identification of not only gene presence but also gene expression and enzymatic activity, thereby improving predictions of *in vivo* outcomes.^168^ ^,^ ^169^ Because results are highly sensitive to methodological and culture conditions, there is a critical need for standardized protocols, shared datasets, and rigorous negative and positive controls (e.g., heat-killed controls, germ-free comparisons).^170^ ^,^ ^171^

### Microbiome role in disease (as cause)

**Figure 1.**
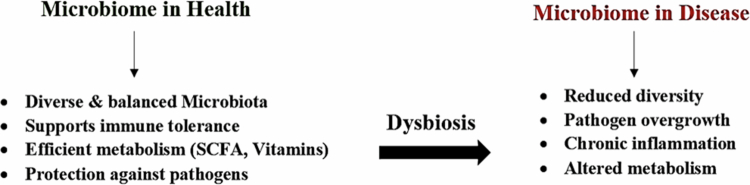
Microbiome mediated modulation of health and disease across human organs.

The human microbiome, comprising trillions of microorganisms inhabiting various body sites, plays a crucial role in maintaining physiological homeostasis and overall health (Figure 1).^147^ Disruption of this intricate microbial ecosystem, commonly referred to as dysbiosis, has emerged as a critical contributor to the development and progression of a wide range of diseases, including gastrointestinal, metabolic, neurological, cardiovascular, autoimmune disorders, and even cancer. Gastrointestinal disorders are among the most well-characterized consequences of dysbiosis. Conditions such as inflammatory bowel disease (IBD) and irritable bowel syndrome (IBS) are frequently associated with reduced microbial diversity and overgrowth of pathogenic species, which collectively compromise gut barrier integrity and promote chronic inflammation, exacerbating disease severity and impairing digestive function.^97^ ^,^ ^172^ Beyond the gut, metabolic diseases are closely linked to microbial dysregulation. Obesity, type 2 diabetes, and metabolic syndrome have been associated with alterations in the gut microbiota that enhance energy extraction from food, modulate host metabolic pathways, and drive systemic inflammation, thereby contributing to disease onset and progression. The gut–brain axis illustrates the profound influence of microbiota on neurological health. Dysbiosis can disrupt neural signaling, neurotransmitter synthesis, and immune-mediated pathways, affecting brain function and behavior. Emerging evidence implicates altered microbial communities in conditions such as autism spectrum disorders, depression, and neurodegenerative diseases, including Alzheimer's and Parkinson's disease.^1^ ^,^ ^172^ Cardiovascular health is similarly modulated by gut microbiota. Microbial metabolites, such as trimethylamine-N-oxide (TMAO), have been linked to atherosclerosis and other cardiovascular conditions, and dysbiosis can enhance the production of such metabolites, increasing cardiovascular risk.^2^ ^,^ ^97^ Autoimmune disorders also reflect the impact of microbial imbalances. Diseases, including rheumatoid arthritis and systemic lupus erythematosus, demonstrate how dysbiosis can modulate immune responses, foster chronic inflammation and contribute to autoimmunity. Certain gut microbes can even influence carcinogenesis; for example, sulfate-reducing bacteria generate hydrogen sulfide, a genotoxic metabolite that can induce DNA damage and promote tumorigenesis, highlighting a direct mechanistic link between microbiota and cancer development. Given the broad implications of dysbiosis, therapeutic strategies aimed at restoring microbial balance are of increasing interest. Interventions such as probiotics, prebiotics, and fecal microbiota transplantation have shown promise in alleviating symptoms and modulating disease progression. Nonetheless, further research is required to fully elucidate the underlying mechanisms and optimize their clinical application.^1^ ^,^ ^2^ ^,^ ^97^

### Disease stage-specific and temporal dynamics of the microbiome

Emerging evidence indicates that microbiome alterations are not static but evolve dynamically across disease initiation, progression, and therapeutic intervention.^173^ Disease stage-specific microbial signatures provide critical insights into pathogenesis and clinical outcomes, often revealing functional and metabolic shifts that are not apparent from cross-sectional analyses alone. In early disease stages, microbial changes may reflect subtle functional impairments, whereas advanced stages are frequently characterized by expansion of pathobionts, loss of microbial resilience, and enrichment of pro-inflammatory and stress-associated pathways.^174^

In IBD, early reductions in butyrate-producing bacteria (e.g., *Faecalibacterium prausnitzii*) impair barrier function and regulatory T-cell responses.^175^ ^,^ ^176^ Disease progression is marked by expansion of *Proteobacteria* utilizing inflammation-derived electron acceptors.^177^ Similarly, CRC exhibits staged microbiome remodeling, with early lesions enriched in genotoxic *pks + E. coli* and *Fusobacterium nucleatum,* ^178^ ^,^ ^179^ while advanced tumors harbor communities adapted to hypoxic and immune-evasive niches.^180^

Stage-dependent microbiome alterations have also been reported in metabolic and neurological disorders. In type 2 diabetes, early dysbiosis involves functional shifts in short-chain fatty acid and branched-chain amino acid metabolism,^181^ whereas advanced disease correlates with increased endotoxemia and systemic inflammation. In neurodegenerative conditions such as Parkinson's disease, early reductions in SCFA-producing capacity precede later-stage enrichment of pro-inflammatory taxa associated with neuroinflammation and disease severity.^182^ ^,^ ^183^ Importantly, therapeutic interventions, including diet, pharmacotherapy, and fecal microbiota transplantation, can further reshape these temporal trajectories, underscoring the microbiome's responsiveness to treatment.

Collectively, these observations highlight that disease stage-specific microbiome signatures offer valuable opportunities for early diagnosis, prognostic stratification, and treatment monitoring. Key microbiome signatures associated with early and late stage of disease are summarized in Table 5. Incorporating longitudinal and functional microbiome analyses will be essential for translating microbiome research into clinically actionable precision medicine strategies.

**Table 5.** Early- and late-stage disease–associated microbiome signatures.

Disease	Early/preclinical stage	Late/advanced stage	Dominant functional shifts	Clinical relevance
Inflammatory bowel disease (IBD)	↓ *Faecalibacterium prausnitzii*; ↓ SCFA producers	↑ Proteobacteria (AIEC *E. coli*); ↓ diversity	Early: ↓ butyrate, impaired Treg induction; Late: ↑ LPS, oxidative stress pathways	Early diagnosis; progression monitoring; response to FMT
Ulcerative colitis	Mild loss of Firmicutes; preserved diversity	Expansion of Proteobacteria; mucosal invasion	Shift from barrier dysfunction to chronic inflammatory metabolism	Stratification for biologic vs microbiome therapy
Colorectal cancer (CRC)	↑ *Fusobacterium nucleatum*, pks⁺ *E. coli*	Enrichment of immune-evasive, hypoxia-adapted taxa	Early genotoxicity; Late immune suppression, altered bile acids	Early detection biomarkers; prognosis
Type 2 diabetes	↓ *Akkermansia muciniphila*; altered SCFA pathways	↑ endotoxin-producing taxa; ↓ microbial diversity	Transition from metabolic dysregulation to systemic inflammation	Risk prediction; therapy optimization
Obesity	↑ energy-harvesting pathways	↑ inflammatory and bile acid metabolism pathways	Shift from caloric extraction to chronic inflammation	Personalized nutrition
Parkinson's disease	↓ SCFA producers; altered tryptophan metabolism	↑ Enterobacteriaceae; ↑ LPS	Early barrier dysfunction; late neuroinflammation	Early biomarkers; disease staging
Alzheimer's disease	↓ anti-inflammatory taxa	↑ LPS- and amyloid-producing bacteria	Progressive neuroinflammation and amyloid aggregation	Risk stratification
Atherosclerosis	↑ microbial TMA-production genes	↑ TMAO-associated taxa	Escalating vascular inflammation	Cardiovascular risk prediction

### Gastrointestinal disorders

Gastrointestinal disorders (GIDs) encompass a spectrum of diseases and functional abnormalities affecting the intestines, stomach, liver, and pancreas.^184^ ^,^ ^185^ These conditions include IBD, such as Crohn's disease and ulcerative colitis, IBS, colorectal cancer, and functional disorders like dyspepsia and gastroesophageal reflux disease. Recent research has increasingly highlighted the critical role of the human gut microbiome in the development and progression of these disorders.^186-188^ The gut microbiome, comprising billions of bacteria, viruses, and fungi, plays a crucial role in immune regulation, nutrient metabolism, and the production of bioactive metabolites. Dysbiosis, or alterations in microbial composition, can disrupt metabolic homeostasis, increase intestinal permeability, and trigger chronic inflammation. For example, IBD patients often exhibit reduced abundance of beneficial species such as *Faecalibacterium prausnitzii*, accompanied by decreased production of SCFAs like butyrate. Butyrate supports intestinal epithelial integrity and modulates immune responses, and its deficiency may exacerbate inflammation.^189^ ^,^ ^190^ In IBS, microbial shifts, including increased Bacteroides and Lachnospiraceae, can alter gut motility, enhance visceral sensitivity, and disrupt the gut–brain axis.^191^ ^,^ ^192^ Interactions between the microbiome, the enteric nervous system, and intestinal immune pathways are therefore central to IBS symptomatology.^92^ Molecular mechanisms underlying gastrointestinal disorders involve the activation of inflammatory pathways, elevated production of pro-inflammatory cytokines, epigenetic modifications, and DNA damage.^189^ ^,^ ^190^ In colorectal cancer, certain microbes generate carcinogenic metabolites, such as nitrosamines, which induce genetic mutations and promote tumor growth. The increased abundance of pathogenic species, such as *Bacteroides fragilis*, can activate the NF-κB pathway and elevate pro-inflammatory cytokines, including IL-6 and TNF-α, thereby contributing to chronic inflammation and pre-cancerous epithelial changes.^191^ ^,^ ^192^ Additional mechanisms include interactions with innate and adaptive immune systems, modulation by microbial metabolites such as SCFAs, and effects on intestinal oxidative balance. SCFAs act via GPR41 and GPR43 receptors to reduce inflammation, whereas pro-inflammatory microbial species can increase free radical production, causing oxidative stress and tissue damage.^92^ ^,^ ^190^ Host genetics further influences GID susceptibility. Mutations in genes such as *NOD2*, *ATG16L1*, and *IL23R* are associated with increased risk of Crohn's disease, as they regulate immune responses to the microbiome, and mutations can lead to uncontrolled inflammation. Interactions between host genetics and gut microbiome thus form a central component of GID pathophysiology. Therapeutic strategies targeting microbiomes are under active investigation. Probiotics can restore microbial balance and enhance the production of beneficial metabolites, whereas prebiotics stimulate the growth of beneficial species and improve gut health. Fecal microbiota transplantation (FMT) has demonstrated efficacy in patients with IBD and *Clostridioides difficile* infection, promoting microbiome restoration.^92^ ^,^ ^191^ Dietary modifications, such as reducing saturated fat intake and increasing dietary fiber, also confer protective effects by mitigating inflammation and supporting gut function.^190^ Emerging evidence highlights the role of the gut–brain axis in gastrointestinal disorders. Microbial alterations can impact neural and hormonal signaling, contributing to anxiety, depression, and heightened gut sensitivity. These complex interactions among the nervous system, immune system, and microbiome underpin chronic gastrointestinal conditions. In summary, gastrointestinal disorders result from a complex interplay among the gut microbiome, host genetics, immune responses, and environmental factors, including diet and stress. A comprehensive understanding of these mechanisms can inform targeted therapies and preventive strategies, and recent studies indicate that microbiome modulation, inflammatory response management, and precise dietary interventions offer substantial therapeutic benefits for patients with IBD, IBS, and colorectal cancer.

### Inflammatory bowel disease (IBD)

Inflammatory bowel disease (IBD) is consistently associated with decreased microbial diversity, a reduction in Firmicutes, particularly *Faecalibacterium prausnitzii* and an expansion of pro-inflammatory Proteobacteria such as *Escherichia coli.* ^87^ ^,^ ^91^ The adherent-invasive *E. coli* (AIEC) strain has been shown to adhere to and invade intestinal epithelial cells, stimulate tumor necrosis factor-alpha (TNF-α) production, and activate NF-κB signaling pathways, thereby promoting chronic mucosal inflammation.^88^ In contrast, *F. prausnitzii* produces SCFAs, including butyrate, which reinforce epithelial barrier integrity and suppress pro-inflammatory cytokine production, indicating a protective function.^89^ At the molecular level, microbial metabolites interact with host immune receptors to modulate immune responses. In IBD, reduced SCFA production impairs T_reg_ function, contributing to exaggerated immune activation.^193^ Additionally, bacterial products such as lipopolysaccharide (LPS) from Proteobacteria engage TLR4-mediated signaling, further driving mucosal inflammation.^3^ The study of the microbiome diversity of IBD 100 patients during one year showed relatively stable core microbiome, however, certain bacterial clusters including *Clostridia* pathobionts, *Faecalibacterium* spp., and other species switch between high/low stable states around flares, with some strains undergoing flare-associated replacement, directly supporting stage-specific, flare-linked microbial signatures.^194^ Additionally, the combined metabolomic and metagenomic analysis revealed that both pediatric and adult patients suffering from IBD has disease-specific microbial signatures, mainly the microbial profiling of *Streptococcus salivarius, E. coli, Ralstonia insidiosa*, *Stenotrophomonas maltophilia*, *Erysipelatoclostridium ramosum*, *Blautia spp*., and *Coprococcus comes,* which can be used as a diagnostic marker for IBD.^195^ These findings underscore the dual role of the gut microbiome in either exacerbating or mitigating inflammatory processes in IBD.

### Ulcerative colitis (UC)

Randomized controlled trials have explored FMT as a therapeutic strategy for ulcerative colitis (UC). A landmark study by Paramsothy et al. demonstrated that intensive, multidonor FMT induced clinical remission in UC patients, accompanied by increased microbial richness and restoration of beneficial taxa, including *Bacteroides* and members of the *Lachnospiraceae* family.^196^ Mechanistically, FMT appears to re-establish microbial-mediated immune tolerance and restore key metabolic outputs, such as SCFAs, which are critical for maintaining colonic epithelial integrity and modulating local immune responses.^197^ The work of Dang et al. identified gut microbial signatures that predict response versus non-response to first-line 5-ASA therapy in UC, highlighting species such as *Faecalibacterium prausnitzii* and *Klebsiella pneumoniae,* and linking specific microbial functions to treatment efficacy.^198^ This study also showed the importance of precision medicine, because microbiome not only changes with disease and FMT, however also can forecast response to standard drugs. Similarly, the recent work of Ahmadi et al. on treatment-resistant UC compared microbiomes of refractory versus newly diagnosed patients and showed distinct compositional and functional patterns, suggesting that targeted microbiome modulation may be useful for the new patients with UC. They found that mainly *Lactobacillus ssp.*, *Bifidobacterium ssp.*, and *Bacteroides ssp,* as well as *E. coli* and *Bifidobacterium* can be key players for new therapeutic strategies in UC.^199^

### Crohn's disease (CD)

Crohn's disease (CD) shares several pathological features with ulcerative colitis (UC) but is distinguished by transmural inflammation. Studies have consistently reported enrichment of Enterobacteriaceae, particularly pathogenic *Escherichia coli* strains, which compromise gut barrier integrity and stimulate Th17-mediated immune responses.^200^ Concurrently, a reduced abundance of beneficial genera, such as *Bifidobacterium*, has been observed. Mechanistically, microbial dysbiosis in CD contributes to Paneth cell dysfunction and altered production of antimicrobial peptides, thereby facilitating microbial invasion and perpetuating mucosal injury.^93^ ^,^ ^201^ ^,^ ^202^ The role of the plant-based diet in the CD patients was discussed in a recent paper, where the 12-week diet increased microbial diversity and expanded beneficial genera, such as *Faecalibacterium, Bacteroides*, and SCFA producers, and reduced fecal calprotectin, which is an important indicator of intestinal inflammation.^203^ The mucose-associated mycobiome and bacteriome found a striking depletion of *Cladosporium sphaerospermum* in CD mucosa, however without changes in fecal level. The experimental study in mice showed that this fungus occupied crypt niches and produces AMP, upregulated epithelial junctions and *Wnt* signaling, which lead to protection against inflammation.^204^ A compartive study of healthy first-degree relatives of CD patients showed that baseline microbial composition predicted future Crohn's disease onset several years before diagnosis. High-risk profiles featured depletion of SCFA-producing taxa and enrichment of specific pro-inflammatory species and pathways, suggesting that dysbiosis in microbial profiling is involved in also in the disease initiation, not only its maintenance.^205^

### Irritable bowel syndrome (IBS)

Although irritable bowel syndrome (IBS) is classified as a functional disorder without overt inflammation, numerous studies have identified altered gut microbial profiles. Notably, reduced abundances of *Lactobacillus* and *Bifidobacterium* and an increased Firmicutes/Bacteroidetes ratio have been reported. Beyond a *Firmicutes/Bacteroidetes* ratio, recent studies show IBS is characterized by reduced overall diversity and specific taxonomic patterns, including increased *Enterobacteriaceae, Ruminococcus, Clostridium, Dorea* and decreased *Faecalibacterium.* ^206^ Mechanistic evidence suggests that microbial metabolites, including methane and hydrogen sulfide, can influence gut motility and visceral sensitivity. In addition, bacterial regulation of serotonin (5-HT) biosynthesis in enterochromaffin cells influences gut–brain signaling, thereby contributing to the manifestation of IBS symptoms.^94^ ^,^ ^95^ ^,^ ^207^ Furthermore, Zhai et al. showed that colonization of germ-free mice with *Ruminococcus gnavus* enhanced catabolism of dietary phenylalanine and tryptophane, which activate race-amine–associated receptor 1 (TAAR1) on enterochromaffin cells and stimulate 5-HT biosynthesis, inducing IBS-D–like diarrhea and visceral hypersensitivity.^208^ This paper supports gut microbes and their by-products influencing the gut–brain axis, barrier functions. and immune responses.

### Recurrent *Clostridioides difficile* infection (CDI)

Recurrent *Clostridioides difficile* infection (CDI) exemplifies the critical role of the gut microbiota in modulating susceptibility to infection. Antibiotic-induced dysbiosis diminishes colonization resistance, permitting pathogenic *C. difficile* to proliferate. Clinical studies have demonstrated that FMT effectively restores microbial diversity, reintroduces protective taxa such as Ruminococcaceae and Bacteroidaceae, and suppresses the overgrowth of *C. difficile*. Mechanistically, secondary bile acids produced by commensal bacteria inhibit *C. difficile* spore germination, highlighting the importance of microbial metabolites in maintaining host defense.^79^ ^,^ ^209^ ^,^ ^210^ Furthermore, recent study of Tian et al. presents a computational pipeline that uses meta-analysis and machine learning to identify microbiome signatures of *C. difficile* resistance and to rationally design a synthetic FMT consortium (sFMT1). It shows that sFMT1 suppresses *C. difficile* virulence *in vitro* and in animal models, and that protection is driven by proline-competing (Stickland-fermenting) strains rather than secondary bile acid metabolism, with a single strain providing protection comparable to human FMT.^211^

### Metabolic diseases

Dysbiosis has been strongly associated with metabolic disorders, including obesity, type 2 diabetes, and metabolic syndrome. Alterations in gut microbial composition can affect energy extraction from the diet, modulate host metabolic pathways, and promote systemic inflammation, thereby contributing to the onset and progression of these diseases.

### Obesity

Obesity has been consistently associated with alterations in gut microbial communities, notably an increased Firmicutes-to-Bacteroidetes ratio.^80^ Human and animal studies indicate that Firmicutes exhibit an enhanced capacity to extract energy from complex polysaccharides, resulting in increased caloric absorption and adiposity.^81^ Furthermore, high-fat diets have been shown to shift the gut microbiome toward pro-inflammatory taxa, such as *Bilophila wadsworthia*, which can promote intestinal inflammation via sulfite reduction. Mechanistically, microbial metabolites, especially SCFAs, exert dual effects: while butyrate generally enhances insulin sensitivity, excess acetate may stimulate lipogenesis through activation of G-protein coupled receptor 46(GPR4).^82^

### Type 2 diabetes (T2D)

Dysbiosis has also been implicated in the pathogenesis of type 2 diabetes (T2D). Patients with T2D frequently exhibit reduced abundance of *Akkermansia muciniphila*, a mucin-degrading bacterium that supports gut barrier integrity. Depletion of *A. muciniphila* increases intestinal permeability, leading to systemic endotoxemia, with LPS activating TLR4-mediated pathways and promoting chronic low-grade inflammation. This inflammatory milieu impairs insulin signaling in peripheral tissues, contributing to insulin resistance. Additionally, enrichment of *Prevotella copri* has been linked to elevated production of branched-chain amino acids (BCAAs), which are inversely associated with insulin sensitivity.^83^ ^,^ ^85^

### Non-alcoholic fatty liver disease (NAFLD)

The gut–liver axis plays a central role in the pathogenesis of non-alcoholic fatty liver disease (NAFLD). Dysbiosis can increase endogenous ethanol production by *Escherichia coli* strains, contributing to hepatocyte injury and hepatic steatosis.^86^ Additionally, microbial metabolites such as trimethylamine (TMA), produced by *Clostridia* and members of the *Enterobacteriaceae*, are converted to trimethylamine-N-oxide (TMAO) in the liver, promoting lipid accumulation and hepatic inflammation.^212^

### Obesity-associated inflammation and cancer risk

Chronic low-grade inflammation associated with obesity is exacerbated by microbial dysbiosis. Endotoxin-producing Gram-negative bacteria can amplify systemic inflammation, increasing circulating cytokines such as IL-1β and TNF-α. These inflammatory mediators contribute to insulin resistance and simultaneously create a pro-carcinogenic environment.^213^ This metabolic–inflammation axis provides a mechanistic link connecting obesity, type 2 diabetes, and heightened cancer susceptibility.

### Microbial interventions

Weight-loss interventions, including dietary modifications, bariatric surgery, and prebiotic supplementation, have been associated with partial restoration of gut microbial diversity and improvements in host metabolic function.^196^ ^,^ ^214^ ^,^ ^215^ Dietary fiber stimulates the growth of butyrate-producing bacteria, thereby enhancing insulin sensitivity and reducing adiposity. FMT has also been investigated as a therapeutic strategy for metabolic syndrome, with early clinical studies reporting improvements in insulin resistance and lipid profiles.^3^

### Neurological disorders

The human gut microbiome plays a pivotal role in neurological health through the gut–brain axis, a bidirectional communication network that encompasses vagus nerve, neuroendocrine signaling, immune pathways, and microbial metabolites. Disruptions in microbial community composition and function collectively termed dysbiosis have been increasingly linked to a broad range of neurological disorders, including Parkinson's disease, Alzheimer's disease (AD), MS, depression, autism spectrum disorder (ASD), and complications following traumatic brain injury (TBI). Emerging evidence suggests that specific microbial taxa and their metabolites modulate neuroinflammation, neuronal signaling, and neurodegenerative processes.^98^

### Parkinson's disease

Parkinson's disease (PD) is closely associated with alterations in gut microbiota, including a reduced abundance of *Prevotella* species and an enrichment of pro-inflammatory taxa, such as Enterobacteriaceae. Clinical studies indicate that patients with PD exhibit elevated levels of LPS-producing bacteria, which promotes systemic inflammation and microglial activation.^8^ ^,^ ^9^ Concurrently, SCFAs, mainly butyrate, are diminished, compromising intestinal epithelial barrier integrity and facilitating translocation of bacterial antigens. This process can enhance α-synuclein misfolding and aggregation in enteric neurons, which may propagate to the central nervous system via vagus nerve.^104^ Collectively, these findings suggest that gut dysbiosis can trigger neurodegeneration through mechanisms involving immune priming and protein misfolding.

### Alzheimer's disease

Alzheimer's disease (AD) has been linked to gut microbiota dysbiosis, characterized by an increased abundance of pro-inflammatory taxa, such as *Escherichia/Shigella*, and a reduction in beneficial species, including *Bifidobacterium* and *Eubacterium rectale*. Dysbiotic microbial communities can elevate systemic levels of LPS and amyloid-like bacterial proteins, which may cross the blood–brain barrier (BBB) and promote amyloid-beta (Aβ) aggregation.^3^ Concurrently, diminished SCFAs impair T_reg_ development, weakening anti-inflammatory control within the central nervous system. Certain microbial metabolites, including trimethylamine-N-oxide (TMAO), may further exacerbate tau hyperphosphorylation and Aβ deposition. Collectively, these findings indicate that microbial dysbiosis contributes to AD pathogenesis through immune dysregulation and metabolic interference.

### Multiple sclerosis

Multiple sclerosis (MS) patients frequently exhibit gut microbiota alterations, including enrichment of *Akkermansia muciniphila* and *Methanobrevibacter smithii*, along with reduced abundance of *Parabacteroides distasonis*. These microbial shifts are associated with pro-inflammatory cytokine profiles and enhanced Th17 cell differentiation.^101^ ^,^ ^102^ Microbiota- SCFAs, particularly propionate, play a key role in promoting T_reg_ expansion, and their deficiency exacerbates autoimmune demyelination. Dysbiosis in MS also compromises intestinal barrier integrity, increasing systemic exposure to microbial antigens and promoting central nervous system-directed autoimmunity. Experimental FMT has shown potential to partially restore immune homeostasis, highlighting a promising therapeutic avenue.^58^ ^,^ ^105^

### Depression and mood disorders

The gut microbiome is increasingly recognized as a critical determinant of mental health. Patients with major depressive disorder (MDD) often exhibit decreased abundances of *Lactobacillus* and *Bifidobacterium* species, which are key producers of GABA and serotonin precursors, as well as other neuroactive compounds; their depletion can reduce central neurotransmitter availability.^106^ Concurrently, enrichment of pro-inflammatory bacteria elevates circulating cytokines, including IL-6 and TNF-α, which can cross the blood–brain barrier and impair synaptic plasticity. Moreover, dysbiosis-driven alterations in tryptophan metabolism shift toward the kynurenine pathway, generating neurotoxic metabolites implicated in depressive symptomatology.^99^

### Autism spectrum disorder

Autism spectrum disorder (ASD) is associated with gut microbiota alterations, including increased abundances of *Clostridium bolteae* and *Desulfovibrio* species, coupled with reductions in *Bifidobacterium* and *Prevotella*. These microbial changes correlate with gastrointestinal symptoms commonly observed in autistic individuals.^106^ Microbial metabolites, notably p-cresol and propionic acid, exert neurotoxic effects by disrupting mitochondrial function, neurotransmission, and neuronal gene expression. In mouse models, transplantation of ASD-associated microbiota has been shown to induce behavioral deficits, providing evidence for a causal role. Preliminary interventions using probiotics and dietary fiber supplementation have demonstrated potential benefits in modulating gut microbial composition and alleviating behavioral symptoms.

### Traumatic brain injury

Emerging evidence underscores the role of the gut microbiota in recovery following traumatic brain injury (TBI). Post-injury dysbiosis is characterized by reduced microbial diversity, depletion of SCFA-producing taxa such as *Faecalibacterium prausnitzii*, and enrichment of pro-inflammatory bacteria.^3^ This microbial imbalance amplifies systemic inflammation, increases blood–brain barrier permeability, and exacerbates neuronal injury. Preclinical studies indicate that FMT can restore eubiosis, resulting in improved neurological outcomes and reduced neuroinflammation.^3^

### Cardiovascular diseases

The human gut microbiome exerts significant influence on cardiovascular health through the production of microbial metabolites, modulation of immune signaling, and regulation of vascular function. Dysbiosis has been strongly associated with several cardiovascular diseases, including atherosclerosis, hypertension, myocardial infarction, and heart failure.

### Atherosclerosis

Atherosclerosis represents one of the most well-characterized links between gut microbiota and cardiovascular disease. Specific bacterial families, including *Enterobacteriaceae* and *Clostridiaceae*, metabolize dietary choline, L-carnitine, and phosphatidylcholine into trimethylamine (TMA), which is subsequently oxidized in the liver to trimethylamine N-oxide (TMAO).^165^ ^,^ ^166^ Elevated TMAO levels promote upregulation of macrophage scavenger receptors (CD36, SR-A1), enhance foam cell formation, and induce vascular inflammation, thereby accelerating atherogenesis. Additionally, bacterial species such as *Bacteroides fragilis* and *Escherichia coli* produce metabolites, including phenylacetic acid and indoxyl sulfate, which contribute to endothelial dysfunction and oxidative stress.^109^

### Hypertension

Hypertension has been closely linked to alterations in SCFA production. Under normal conditions, commensal bacteria such as *Lactobacillus*, *Bifidobacterium*, and members of the Firmicutes phylum ferment dietary fibers to produce acetate, butyrate, and propionate. These SCFAs act on G-protein–coupled receptors (GPR41, GPR43), promoting vasodilation and exerting anti-inflammatory effects that contribute to blood pressure regulation.^110^ ^,^ ^111^ In hypertensive patients, dysbiosis reduces the abundance of SCFA-producing bacteria, diminishing T_reg_ expansion and impairing endothelial nitric oxide (NO) signaling. Concurrently, increased levels of *Prevotella* and *Klebsiella* elevate circulating endotoxins (LPS), which activate TLR4-mediated pathways and promote chronic vascular inflammation.

### Myocardial infarction (MI)

The severity and outcomes of myocardial infarction (MI) are also influenced by gut microbiota. Plasma metabolomic analyses indicate that elevated indole- and phenyl-derived metabolites, produced by *Bacteroides* and *Clostridium* species, are associated with poorer postoperative recovery and an increased risk of adverse cardiovascular events following vascular interventions.^109^ Mechanistically, these microbial metabolites impair endothelial repair, enhance platelet aggregation, and exacerbate systemic inflammation, collectively aggravating ischemic injury.^200^ ^,^ ^216^

### Heart failure

Emerging evidence increasingly highlights the role of gut microbiota in the pathophysiology of heart failure. Patients with heart failure often exhibit dysbiosis characterized by expansion of *Enterococcus faecalis* and members of the *Proteobacteria* phylum, taxa capable of translocating across a compromised intestinal barrier.^111^ This microbial translocation contributes to low-grade endotoxemia, triggering systemic cytokine release, including IL-6 and TNF-α, and promoting maladaptive cardiac remodeling. Furthermore, elevated levels of trimethylamine-N-oxide (TMAO) in these patients are associated with worse prognosis and increased mortality.^2^ Collectively, these findings suggest that gut microbiota influence heart failure progression through intertwined mechanisms of metabolic dysregulation, via TMAO and LPS, and immune activation, ultimately exacerbating myocardial dysfunction.^84^ ^,^ ^217^

### Autoimmune diseases

The human microbiome plays a central role in maintaining immune tolerance, and emerging evidence implicates dysbiosis as a critical contributor to autoimmune diseases. Alterations in microbial composition can perturb immune signaling, amplify pro-inflammatory pathways, and compromise self-tolerance, thereby promoting autoimmune responses. Distinct microbial signatures and associated molecular mechanisms have been identified in several autoimmune conditions, including systemic sclerosis, type 1 diabetes, and inflammatory bowel disease, highlighting the integral role of the microbiome in disease pathogenesis.

### Systemic sclerosis

Systemic sclerosis (SSc) is a complex autoimmune disorder marked by fibrosis, vasculopathy, and immune dysregulation. Patients with SSc frequently exhibit profound intestinal dysbiosis, including reduced abundances of commensal taxa such as *Faecalibacterium prausnitzii* and *Clostridia*, alongside enrichment of *Fusobacterium* and *Prevotella* species.^112^ These microbial alterations promote enhanced Th17 cell differentiation while impairing T_reg_ function, resulting in elevated IL-17 and IL-6 production that drives systemic inflammation and tissue fibrosis. Additionally, diminished SCFA production compromises epithelial barrier integrity, facilitating microbial translocation and perpetuating immune activation.

### Type 1 diabetes (T1D)

In type 1 diabetes (T1D), the gut microbiota has been implicated in the breakdown of peripheral tolerance, facilitating autoreactive T-cell activation and subsequent destruction of pancreatic β-cells. Metagenomic analyses reveal a reduction in butyrate-producing bacteria, including *Bifidobacterium adolescentis* and *Roseburia*, accompanied by increased abundance of *Bacteroides* species.^114^ These microbial alterations are associated with decreased SCFA levels, which weaken mucosal immunity and impair T_reg_ expansion.^113^ Dysbiosis further enhances antigen-presenting cell (APC) activation, promoting IL-12 and IFN*-*γ secretion that drives autoreactive CD8⁺ T-cell responses against pancreatic islets. Additionally, reduced microbial diversity correlates with increased intestinal permeability (“leaky gut”), allowing bacterial LPS to enter systemic circulation and amplify autoimmune cascades.^113^ ^,^ ^114^

### Inflammatory bowel disease (IBD) and related autoimmunity

Although inflammatory bowel disease (IBD) is primarily a gastrointestinal disorder, it exhibits pronounced autoimmune characteristics driven by interactions between the microbiota and the host immune system. Patients with Crohn's disease and ulcerative colitis frequently show enrichment of adherent-invasive *Escherichia coli* (AIEC) and depletion of beneficial *Clostridium* XIVa clusters.^81^ Mechanistically, these microbes activate NF-κB signaling in intestinal epithelial cells, resulting in the excessive production of IL-1β, TNF-α, and IL-23. This establishes a chronic pro-inflammatory loop that amplifies Th17 and Th1 responses while impairing mucosal tolerance. Furthermore, microbial metabolites such as hydrogen sulfide and phenolic compounds directly damage the intestinal epithelium, exacerbating inflammation and perpetuating autoimmune-driven pathology.^96^

### HIV infection and the microbiome

HIV infection profoundly reshapes the gut and vaginal microbiomes, contributing to accelerated disease progression. Depletion of gut CD4⁺ T cells compromises epithelial barrier integrity, facilitating microbial translocation of LPS and microbial DNA, which drives systemic immune activation even in individuals receiving antiretroviral therapy (ART).^218^ Gut dysbiosis in HIV is characterized by reduced *Bacteroides* and enrichment of *Prevotella*, leading to Th17 cell loss and impaired mucosal defense.^219^ In the vaginal microbiome, loss of *Lactobacillus crispatus* and dominance of *Gardnerella vaginalis* exacerbate genital inflammation and increase susceptibility to HIV infection.^220^ Dysbiosis also diminishes the abundance of SCFA-producing taxa, thereby weakening T_reg_ differentiation and mucosal homeostasis. Microbial metabolites further enhance Toll-like receptor (TLR)-driven interferon responses, promoting T-cell exhaustion. Collectively, these interactions establish a vicious cycle in which HIV-induced dysbiosis accelerates immune dysfunction, thereby amplifying disease progression.

### Cancer

The gut microbiome has emerged as a pivotal determinant of cancer initiation, progression, and therapeutic response. Dysbiosis can perturb host metabolism, compromise immune surveillance, and influence epigenetic regulation, collectively fostering a tumor-promoting microenvironment. Distinct microbial profiles and mechanistic pathways have been implicated in several cancers, including colorectal cancer, hepatocellular carcinoma, gastric cancer, and oral malignancies, highlighting the integral role of microbiota in oncogenesis and cancer therapy outcomes.

### Colorectal cancer (CRC)

Colorectal cancer (CRC) is the most extensively investigated malignancy in relation to gut microbiomes. A defining feature of CRC-associated dysbiosis is the enrichment of *Fusobacterium nucleatum* and enterotoxigenic *Bacteroides fragilis* (ETBF).^221^ *F. nucleatum* promotes tumorigenesis by adhering to epithelial cells via its FadA adhesin, which activates β-catenin signaling, stimulating proliferation and inhibiting apoptosis. Additionally, it recruits tumor-infiltrating myeloid cells that produce IL-6 and TNF-α, sustaining chronic inflammation. ETBF contributes through secretion of *B. fragilis* toxin (BFT), which cleaves E-cadherin, disrupts cell–cell adhesion, and activates NF-κB signaling, thereby enhancing IL-17–mediated inflammation.^222^ Other microbial contributors include genotoxin-producing *Escherichia coli* harboring the pks island, which generates colibactin, a DNA-alkylating agent that induces double-strand breaks and genomic instability.^96^

### Hepatocellular carcinoma (HCC)

The gut microbiome also plays a critical role in hepatocellular carcinoma (HCC) via dysregulation of the gut–liver axis. Dysbiosis, characterized by expansion of *Enterococcus faecalis* and *Proteobacteria*, increases intestinal permeability and facilitates translocation of LPS.^223^ LPS activates Toll-like receptor 4 (TLR4) on hepatic Kupffer cells, triggering the release of IL-6, TNF-α, and reactive oxygen species (ROS), which collectively drive hepatocyte injury, fibrosis, and tumorigenesis. Moreover, microbial metabolism of bile acids is altered, leading to elevated levels of deoxycholic acid (DCA), a metabolite that induces DNA damage and senescence-associated secretory phenotypes (SASP), further promoting carcinogenesis. Emerging evidence also suggests that the gut microbiome influences responses to PD-1–based immunotherapy in HCC patients, underscoring its role in modulating both tumor progression and therapeutic efficacy.^224^

### Gastric cancer

*Helicobacter pylori* remains the principal microbial driver of gastric cancer; however, other components of the gastric microbiota also contribute to tumorigenesis. Dysbiosis in patients with premalignant lesions is characterized by enrichment of *Lactobacillus reuteri* and *Streptococcus anginosus.* ^81^ These bacteria produce excessive lactic acid, which functions as an oncometabolite, promoting angiogenesis and tumor growth. Concurrently, microbial dysbiosis amplifies chronic gastritis through elevated IL-1β and IL-8 production, fostering a pro-tumorigenic microenvironment that facilitates cancer progression.

### Oral cancers

The oral microbiome has emerged as an important contributor to oral squamous cell carcinoma (OSCC). Patients with OSCC frequently exhibit elevated levels of *Porphyromonas gingivalis* and *Fusobacterium nucleatum.* ^225^ *P. gingivalis* secretes gingipains that degrade E-cadherin and activate β-catenin signaling, driving uncontrolled epithelial proliferation. It also modulates the tumor immune microenvironment by upregulating PD-L1 expression on epithelial cells, facilitating immune evasion. Likewise, *F. nucleatum* promotes IL-6-mediated STAT3 activation, enhancing epithelial–mesenchymal transition (EMT) and increasing metastatic potential.

### Microbiome as therapy

The human microbiome constitutes a highly complex and dynamic ecosystem of microorganisms that continuously interact with their host, playing an essential role in maintaining metabolic, immune, and physiological homeostasis.^10^ Increasing evidence links microbiome dysbiosis to a broad spectrum of diseases, including gastrointestinal, metabolic, immune, and neurological disorders, providing a compelling rationale for microbiome-targeted therapeutic approaches.^189^ ^,^ ^226^ The concept of microbiome-based therapy rests on the premise that targeted modulation of microbial composition or function can influence pathophysiological processes and restore health.^227^ This paradigm encompasses a wide array of interventions, ranging from FMT, which is the most clinically established application,^228^ to probiotics, prebiotics, postbiotics, and engineered live biotherapeutics designed for specific therapeutic purposes.^229^ ^,^ ^230^ A defining feature of this emerging field is its interdisciplinary nature. Advances in metagenomics, metatranscriptomics, and metabolomics have enabled precise characterization of microbial composition and metabolic activity,^231^ facilitating the development of personalized therapeutic strategies tailored to individual microbiome profiles.^232^ Despite its promise, several challenges remain. Inter-individual variability in microbiome composition, difficulties in standardizing interventions, uncertainties regarding long-term safety, and regulatory hurdles represent major barriers to widespread clinical implementation.^77^ ^,^ ^233^ Furthermore, current evidence often demonstrates correlations rather than causation, highlighting the need for rigorous studies to clarify the mechanistic links between microbial alterations and disease.^234^ Overall, the microbiome is increasingly recognized as both a novel therapeutic target and a pivotal component of precision medicine. Future integration of basic science, multi-omics technologies, and clinical research is expected to enable interventions that not only manage disease but also promote long-term health by restoring ecological balance within the host.

### Inflammatory bowel disease (IBD)

Inflammatory Bowel Disease (IBD), encompassing Crohn's disease and ulcerative colitis, is fundamentally characterized by a dysregulated immune response directed against the intestinal microbiota in genetically predisposed individuals. Central to its pathophysiology is microbial dysbiosis, defined by a marked reduction in microbial diversity.^1^ ^,^ ^3^ This imbalance involves depletion of commensal bacteria with anti-inflammatory properties, such as *Faecalibacterium prausnitzii*, a major producer of SCFAs,^1^ ^,^ ^17^ alongside expansion of pro-inflammatory pathobionts. Notably, AIEC strains exemplify this shift; these microbes can invade intestinal epithelial cells, persist within macrophages, and sustain chronic inflammation through NF-κB activation and subsequent release of pro-inflammatory cytokines, including TNF-α and IL-6.^3^ ^,^ ^5^ The resulting deficiency in SCFAs, particularly butyrate, exacerbates disease pathology by compromising epithelial barrier integrity, impairing T_reg_ differentiation, and increasing intestinal permeability, thereby perpetuating a self-reinforcing cycle of immune activation and inflammation. Insights into these mechanisms have guided the development of microbiome-targeted therapies. FMT has emerged as a promising intervention, restoring microbial diversity and demonstrating efficacy in inducing both endoscopic and clinical remission in ulcerative colitis.^235^ Concurrently, next-generation probiotics comprising defined bacterial consortia or specific strains such as *E. coli* Nissle 1917 offer a more precise approach. These beneficial microbes act through competitive exclusion of pathobionts, enhancement of mucosal barrier function, and modulation of host immune responses.^3^ ^,^ ^236^ Prebiotics and dietary interventions, including low FODMAP or high-fiber regimens, provide selective substrates that promote the growth and activity of beneficial bacteria, indirectly supporting a healthier microbiota composition.^1^ Additionally, postbiotics, involving the administration of microbial-derived metabolites such as SCFAs, offer a novel strategy to harness the anti-inflammatory and barrier-enhancing effects of the microbiome without the need for live organisms.^17^ Collectively, these microbiome-centric therapies signify a paradigm shift in IBD management, moving beyond broad immunosuppression towards strategies that restore intestinal homeostasis and re-establish host–microbiota equilibrium.^1^ ^,^ ^3^ ^,^ ^235^

### Metabolic disorders (obesity & type 2 diabetes)

Human gut microbiota has emerged as a central regulator of metabolic homeostasis, exerting profound effects on the development and progression of obesity and type 2 diabetes (T2D).^2^ ^,^ ^16^ By extracting energy from otherwise indigestible dietary components, modulating bile acid metabolism, and influencing systemic low-grade inflammation, the gut microbial community orchestrates key aspects of host metabolism.^237^ Dysbiosis, a disruption in this intricate ecosystem, can precipitate metabolic endotoxemia, characterized by increased intestinal permeability and translocation of bacterial LPS into the circulation. This process triggers chronic inflammation via Toll-like receptor 4 (TLR4) signaling, ultimately impairing insulin sensitivity and glucose homeostasis.^14^ ^,^ ^18^ A critical component of this metabolic regulation is the production of SCFAs by commensal bacteria such as *Faecalibacterium prausnitzii* and *Roseburia* spp. SCFAs reinforce epithelial barrier integrity, stimulate glucagon-like peptide-1 (GLP-1) secretion, and modulate hepatic gluconeogenesis, thereby maintaining metabolic balance.^2^ ^,^ ^17^ In dysbiosis, the depletion of these beneficial taxa reduces SCFA availability, exacerbating barrier dysfunction, inflammation, and metabolic dysregulation. Several microbiome-targeted interventions have shown promise in mitigating metabolic disorders. Probiotics and synbiotics, particularly strains of *Lactobacillus* (e.g., *L. acidophilus*, *L. casei*) and *Bifidobacterium* (e.g., *B. breve*, *B. longum*), have demonstrated consistent benefits in improving insulin sensitivity, reducing body mass index (BMI), and decreasing waist circumference in clinical studies. These effects are largely attributed to enhanced intestinal barrier function, reduced LPS translocation, and modulation of pro-inflammatory pathways.^20^ ^,^ ^238^ ^,^ ^239^ Personalized nutrition represents another innovative avenue, integrating microbiome profiling with clinical and dietary data through machine learning algorithms to predict individual glycemic responses. Such approaches enable tailored dietary interventions that optimize metabolic outcomes according to an individual's unique microbial signature.^21^ ^,^ ^237^ Targeted bacteriotherapy has also gained traction, exemplified by supplementation with *Akkermansia muciniphila*. Both live and pasteurized forms of this mucin-degrading bacterium have been shown in human trials to enhance insulin sensitivity, reduce systemic inflammation, and improve lipid profiles, highlighting its potential as a next-generation microbial therapeutic for metabolic diseases.^240^ Many research studies indicated that gut microbiota constitutes a pivotal interface linking diet, metabolism, and immune function. Therapeutic strategies that restore microbial balance through probiotics, personalized nutrition, or targeted microbial supplementation offer a novel and complementary approach to conventional management of obesity and T2D. Future investigations should prioritize large-scale randomized controlled trials and mechanistic studies to validate these interventions and facilitate their integration into clinical practice.^2^ ^,^ ^16^ ^,^ ^237^

### Neurological & psychiatric disorders (the gut–brain axis)

The gut–brain axis constitutes a sophisticated, bidirectional communication network that links the gastrointestinal tract with the central nervous system through neural, endocrine, and immune pathways.^4^ ^,^ ^18^ At the core of this interplay is the gut microbiota, which actively produces an array of neuroactive compounds, including GABA, serotonin, dopamine, and SCFAs.^241^ ^,^ ^242^ These microbial metabolites can influence central nervous system function directly, crossing the blood-brain barrier to modulate neuroinflammation, neurogenesis, synaptic plasticity, and behavior.^4^ ^,^ ^22^ Dysbiosis perturbations in microbial composition or function have been increasingly implicated in the pathophysiology of a spectrum of neurological and psychiatric disorders. It is associated with hyperactivity of the hypothalamic-pituitary-adrenal (HPA) axis, exaggerated stress responses, and persistent neuroinflammatory processes, hallmark features observed in major depressive disorder, anxiety disorders, and autism spectrum disorder (ASD).^4^ ^,^ ^18^ ^,^ ^23^ Mechanistic insights into the gut–brain axis have catalyzed the development of microbiome-targeted therapeutic strategies. Psychobiotics live microorganisms that confer mental health benefits when administered in adequate amounts have shown clinical promise. Strains such as *Lactobacillus helveticus* R0052 and *Bifidobacterium longum* R0175 have been demonstrated to reduce cortisol levels, attenuate systemic inflammation, and improve subjective measures of anxiety and depression.^241^ ^,^ ^242^ Dietary interventions represent another accessible approach; Mediterranean-style diets, rich in fiber, polyphenols, and unsaturated fatty acids, promote a favorable microbial profile characterized by enhanced diversity and SCFA production, which correlates with improvements in mood and cognitive performance.^22^ ^,^ ^241^ FMT is emerging as a potential therapeutic modality for ASD, with early-phase studies indicating improvements not only in gastrointestinal symptoms but also in behavioral outcomes, underscoring the causal influence of the microbiome on neurological function.^18^ ^,^ ^23^ In summary, modulating the gut–brain axis through psychobiotics, dietary strategies, and microbial restoration therapies holds significant promise for influencing the trajectory of neurological and psychiatric disorders. Future research should prioritize mechanistic elucidation, standardized intervention protocols, and large-scale randomized controlled trials to rigorously assess efficacy and enable translation into clinical practice.^4^ ^,^ ^18^ ^,^ ^241^

### Autoimmune diseases (rheumatoid arthritis & multiple sclerosis)

The gut microbiome plays a central role in shaping and regulating the host immune system, particularly by maintaining the delicate balance between pro-inflammatory T-helper 17 (Th17) cells and anti-inflammatory T_regs_. In autoimmune diseases such as rheumatoid arthritis (RA) and MS, microbial dysbiosis can disrupt this equilibrium, fostering a Th17-dominant environment that drives pathogenic autoimmune activation.^1^ ^,^ ^17^ ^,^ ^24^ This dysregulation is further amplified by mechanisms including molecular mimicry where microbial antigens cross-react with host self-antigens and increased intestinal permeability (“leaky gut”), which permits translocation of microbial products into systemic circulation, perpetuating chronic inflammatory responses. Therapeutic strategies targeting gut microbiota have shown promise in modulating autoimmune disease pathways. Promoting T_reg_ differentiation via probiotics, prebiotics (e.g., inulin), and synbiotics is a key approach. These interventions selectively enrich SCFA-producing bacteria, which enhance T_reg_ proliferation and function, thereby suppressing aberrant immune activation.^1^ ^,^ ^17^ ^,^ ^235^ Restoration of intestinal barrier integrity represents another critical strategy; targeted interventions ranging from specific probiotics and postbiotics to dietary modifications reduce microbial translocation and attenuate systemic inflammation. Moreover, microbiome modulation may complement conventional therapies. For instance, optimizing gut microbial composition can enhance the efficacy of immunomodulatory drugs such as methotrexate while potentially reducing adverse effects, through mechanisms involving microbial metabolism of pharmaceuticals and modulation of host immune pathways.^24^ ^,^ ^235^

### Microbiome-regulated molecular pathways and their role in disease

Microbial metabolites and toxins modulate host biology through a range of molecular pathways that extend far beyond simple compositional shifts in the gut microbiome. Among the most consequential are genotoxic pathways, in which bacterial products directly damage host DNA, drive genomic instability, impaired barrier function, and ultimately contributing to inflammation and carcinogenesis- Inter-strand cross-links (ICLs) are formed in infected cells because of Colibactin alkylates deoxyadenosines within AT- rich motifs. DNA replication is damaged by ICLs and needs repair in a replication- coupled manner. Genomic instability is produced because of induction of double-strand breaks in DNA. An irreversible cell cycle arrests occurred and followed by apoptosis as the load of toxigenic bacteria is increased.^243^ On the other hand, cell repairing is high at lower densities but not all damage authentically. Errors in repair generate chronic mitotic and chromosomal aberrations are generated together by errors in repair with a high frequency of gene mutation ascending seemingly through translesion synthesis past the Coli-bactin-ICL remnant. A research work using neonatal rat pups born to mothers fed commensal *pks* + *E. coli* indicated high damage of DNA double-strand in epithelial cells of gut during the first week of life compared with pups originating from mothers treated with a non-producing *E. coli clbA*- mutant. Perturbed intestinal homeostasis was associated with Persistence of pks + bacteria into adulthood modal, including altered apoptosis patterns, proliferation, and differentiation, and high permeability of the gut. The tricyclic pyr- rolobenzodiazepine (PBD) tilimycin leads to alkylation of DNA in cultured cells of human and enterocytes of mice colonized with til + *K. oxytoca*. The response of DNA damage is activated by adduct formation and repair is facilitated particularly through the transcription-coupled nucleotide excision repair pathway. The mutations occurred in repair are mainly single base substitutions (SBS) in guanines lined through purines (Pu-G-Pu), composed with the suitable sites of binding of the canonical PBDs produced through Streptomyces spp. Similarly, both insertions and deletions were also seen in specific genes of cultured human cells. An indolimine-secreting *M. morganii* isolate articulating active aspartate aminotransferase but not an at incomplete mutant damaged DNA substrates in cell-free assays. The phosphorylated histone H2AX (γH2AX) foci is formed as a result of exposure of cultured HeLa cells to indolimines, which is considered as a very sensitive biochemical marker for DNA double- strand breaks.^244^ ^,^ ^245^ Moreover, a study of Jans et al. shows that the oncogenic effect of *pks + E. coli in* colorectal cancer depends on its ability to adhere tightly to intestinal epithelial cells via the FimH and FmlH pilus adhesins. Pharmacological blocking of FimH markedly reduces colibactin-mediated DNA damage and tumor exacerbation, and allelic variation in FimH can even confer genotoxic gain-of-function to a probiotic strain. By bringing colibactin production into close proximity with host cells, adhesin-mediated binding enables DNA damage that promotes CRC, highlighting anti-adhesive strategies as a potential way to mitigate colibactin-driven carcinogenesis, especially in high-risk individuals.^246^

### Oncogenic potential

Mutational burden is increased substantially by colorectal cancers compared to normal cells, relatively. By recurrently infuriating low-grade damage of DNA at the enterocyte level commensal genotoxic metabolites may thus endorse following somatic genetic change resulting to CRC. However, the role is poorly understood regarding the duration and timing of commensal genotoxin experience in determining the risk of CRC. Pöltl et al.^247^ summarized in conventional C57BL/6J mice the transitory growth of genotoxic *Klebsiella* reported in patients during the therapy of antibiotic. CRC risk mutations can occur due to sufficient exposure to pks + *E. coli,* which results in changes in growth factor dependence and cell culture differentiation models. Preclinical studies demonstrated an increase in tumor burden with common pks + *E. coli* strains compared to non-producing control strains, using genetically susceptible mouse models.^248^

### Metabolite—microbe interactions

The direct damaging activity of DNA of such bacterial metabolites group reinforced the hypothesis that their existence in the gut could have strong effects on the community of microbes. Additionally, it stands to reason that antagonism of bacterial competitors indicates their real evolutionary tenacity. Two types of competition are seen according to microbiota composition: exploitative competition that follows resource consumption indirectly, and interference competition, whereby one individual is harmed directly another. Other bacteria are antagonized directly by commensal genotoxins; thus, in a biological perspective they are important for interference competition. It is spontaneous that several factors will manage how successfully a genotoxic metabolite firms the enteric microbial community. Determinants of their efficacy are; include the size and distribution of the producing population, delivery mechanism of toxin, and the stability, quantity, and spatial range of released genotoxins.^249^

A contact-dependent system is used to deploy colibactin, thus cells cannot be killed at a distance but may be highly effective at short distances. Mixed species wound infections provide precedence, where an uropathogenic strain of *E. coli* was shown to employ the delivery of toxin to antagonize the survival and growth of *Staphylococci*. *Staphylococcus aureus* is killed by colibactin via causing irreparable DNA damage. The producers benefit from the colibactin antibacterial activities in developing and expanding their niche in the human gut ecosystem. Such function may play an important role especially in the initial colonization of infants. Tronnet et al.^250^ measured colibactin-producing microbes effects on de novo gut microbiome assembly in murine neonates following the transmission of a mother-to-pup of a pks + extra-intestinal pathogenic strain of *E. coli* or its non-genotoxic mutant. Colibactin-dependent phylum-level changes were observed and there was a drop in entire profusion within the first weeks of life. A mouse isolate of pks + *E. coli* exhausted *B. fragilis* and *Enterococcus spp* in the murine intestine of adult and colibactin seems to precisely target *B. fragilis* in human carriers.^251^

### Microbial countermeasures to genotoxicity

The til and pks genomic islands, like many antibacterial toxin systems, encode self-immunity determinants that limit collateral damage to the producer's own genome. Their products act intracellularly to restrict genotoxicity, for example by activating late-stage structural intermediates in the periplasm (ClbP), inactivating the genotoxin itself (ClbS), enhancing efflux of toxic metabolites from til + *Klebsiella* via transporters such as MfsX, or expanding the DNA repair capacity of tilimycin-producing strains through dedicated repair enzymes such as UvrX.^252^ Likewise, ecological competition with genotoxin-producing organisms can select for the emergence of protective traits in susceptible bystander bacteria. The acquisition of “orphan” immunity genes that neutralize small-molecule antimicrobials not produced by the host strain appears to represent a general strategy enabling diverse microbial communities to mitigate antagonism; expression of such resistance determinants has been shown to protect heterologous hosts from the genotoxicity of the cognate metabolite.^252^ ^,^ ^253^

Indole and its derivatives participate in interspecies, intraspecies, and interkingdom signaling within microbial ecosystems. The ability to generate indoles from tryptophan via tryptophanase is widespread among gut bacteria, particularly *Bacteroides* spp. Fluctuations in indole availability modulate the expression of virulence factors in several intestinal pathogens, and indole production has been shown to reduce tilimycin secretion in the murine gut. Specifically, indole diminishes transcript levels of the til biosynthetic genes *npsA* and *npsB*, and simultaneously enhances the conversion of tilimycin to tilivalline, a potent agonist of the host pregnane X receptor that induces the expression of detoxification genes under its control.^254^ Thus, microbiota-derived indole is an effective countermeasure to the enterotoxicity of til + bacteria. Likely, microbiota-derived indolimines are direct ligands for another xenobiotic receptor stated in the intestinal tract, the aryl hydrocarbon receptor (AHR).^255^ Activation of this environmental sensor promotes transcription of AHR target genes involved in xenobiotic metabolism and upregulates IL-6 expression when combined with cytokine stimulation in cultured human cells. Such AHR-mediated responses may support the maintenance of intestinal homeostasis in the presence of indolimine-producing bacteria.^256^

### CD8⁺ T cells and antitumor immunity

CD8⁺ cytotoxic T lymphocytes (CTLs) are central mediators of antitumor immunity in colorectal cancer, and their abundance and functional state strongly predict patient prognosis and response to immunotherapy. Microbiome-derived metabolites have emerged as key regulators of CD8⁺ T-cell metabolism and effector function. Short-chain fatty acids, particularly butyrate and propionate, enhance CD8⁺ T-cell memory formation and cytotoxic capacity by promoting mitochondrial oxidative metabolism and epigenetic remodeling through histone deacetylase (HDAC) inhibition. These effects support sustained interferon-γ (IFN-γ) and granzyme B production within the tumor microenvironment.

Conversely, dysbiosis-associated metabolites can suppress CD8⁺ T-cell–mediated tumor control. *Fusobacterium nucleatum*, a CRC-enriched pathobiont, has been shown to impair antitumor immunity both directly and indirectly. Its metabolites, including formate, contribute to metabolic competition within the tumor niche, limiting nutrient availability for infiltrating CD8⁺ T cells and thereby reducing their effector function. In parallel, *F. nucleatum*–associated signaling promotes immune checkpoint expression, further dampening cytotoxic T-cell activity.^257-259^

### Th17 cells: inflammation, plasticity, and tumor promotion

Th17 cells play a dual role in colorectal cancer, contributing to both host defense and tumor-promoting inflammation depending on context. Microbiome-derived metabolites critically regulate Th17 differentiation and stability. Reduced production of butyrate and other SCFAs diminishes regulatory T-cell (Treg) control, thereby favoring Th17 expansion. At the same time, metabolites produced by CRC-associated bacteria promote sustained IL-17A production, which drives tumor growth by enhancing angiogenesis, recruiting myeloid-derived suppressor cells, and activating pro-oncogenic STAT3 signaling in epithelial cells.^260^

Notably, enterotoxigenic *Bacteroides fragilis* and *F. nucleatum* indirectly promote Th17 polarization through metabolite-mediated epithelial stress responses and cytokine release, reinforcing a chronic inflammatory microenvironment that supports tumor progression.^261^ ^,^ ^262^

### Macrophage polarization and immune suppression

Tumor-associated macrophages (TAMs) are highly responsive to microbial metabolites and represent a major determinant of immune tone in CRC. SCFAs generally promote anti-inflammatory macrophage phenotypes via HDAC inhibition and GPR signaling, supporting tissue repair and immune regulation. In contrast, dysbiosis-derived metabolites favor polarization toward immunosuppressive, tumor-promoting macrophages characterized by elevated IL-10, TGF*-*β, and arginase-1 expression.^263^

*Fusobacterium nucleatum*–derived metabolites, including 5-aminovaleric acid, have been implicated in reprogramming macrophage metabolism and function, skewing TAMs toward a pro-tumorigenic phenotype that suppresses CD8⁺ T-cell activation and supports immune evasion. This macrophage-driven suppression represents a key mechanism by which microbial metabolites indirectly inhibit adaptive antitumor immunity.^264^ ^,^ ^265^

### Microbiome-derived metabolites as mechanistic drivers of disease

Microbiome-derived metabolites represent a critical interface between microbial ecology and host physiology, acting as signaling molecules that directly modulate immune responses, epithelial integrity, metabolism, and epigenetic regulation. While short-chain fatty acids (SCFAs) remain among the most extensively studied microbial metabolites, their biological effects extend well beyond general anti-inflammatory activity and involve well-characterized molecular mechanisms.^266^

### Epigenetic and signaling functions of SCFAs

Among SCFAs, butyrate has emerged as a key epigenetic regulator through its ability to inhibit class I and II histone deacetylases (HDACs). HDAC inhibition by butyrate leads to increased histone acetylation, chromatin relaxation, and transcriptional activation of genes involved in immune tolerance, including *FOXP3*, thereby promoting regulatory T-cell differentiation. In intestinal epithelial cells, butyrate-mediated HDAC inhibition enhances expression of tight junction proteins and antimicrobial peptides, reinforcing barrier integrity and limiting inflammatory signaling. Beyond epigenetic effects, SCFAs signal through G-protein–coupled receptors (GPR41, GPR43, and GPR109A), coordinating metabolic and immune responses in a context-dependent manner.^267^

### Beyond SCFAs: emerging pathogenic microbial metabolites

Recent discoveries have highlighted the pathogenic potential of non-SCFA metabolites produced by disease-associated microbes. In colorectal cancer, *Fusobacterium nucleatum* has been shown to generate metabolites that actively promote tumor progression and immune evasion. Formate, a major end-product of *F. nucleatum* metabolism, supports tumor cell proliferation by fueling one-carbon metabolism and enhancing nucleotide biosynthesis. Elevated intratumoral formate levels have been associated with increased tumor growth and metabolic plasticity.

In addition, *F. nucleatum* produces 5-aminovaleric acid, a lysine-derived metabolite that interferes with host amino acid metabolism and modulates immune responses within the tumor microenvironment. This metabolite has been implicated in suppressing antitumor immune surveillance and promoting a pro-tumorigenic niche by altering macrophage and T-cell function. Collectively, these metabolites provide a direct biochemical link between microbial dysbiosis and oncogenic processes.^268^ ^,^ ^269^

### Conclusion

The microbiome should be regarded as an integral component of human biology and one of the important key interfaces between genetics, environment, and diseases. Incorporating microbiome profiling into clinical decision-making can significantly improve disease prevention, refining diagnosis, and personalizing therapy. Future advances will depend on standardized multi-omics approaches, longitudinal studies, and carefully designed clinical trials that translate mechanistic insights into safe and effective interventions. Safeguarding microbial diversity and function is therefore not only essential for maintaining health but also foundational to the evolution of precision and systems-based medicine.

## Data Availability

Not applicable.
